# Evolution of Oxygen Vacancy Sites in Ceria-Based High-Entropy Oxides and Their Role in N_2_ Activation

**DOI:** 10.1021/acsami.3c16521

**Published:** 2024-04-29

**Authors:** Omer Elmutasim, Aseel G. Hussien, Abhishek Sharan, Sara AlKhoori, Michalis A. Vasiliades, Inas Magdy Abdelrahman Taha, Seokjin Kim, Messaoud Harfouche, Abdul-Hamid Emwas, Dalaver H. Anjum, Angelos M. Efstathiou, Cafer T. Yavuz, Nirpendra Singh, Kyriaki Polychronopoulou

**Affiliations:** † Mechanical Engineering Department, Khalifa University, P.O. Box 127788, Abu Dhabi, United Arab Emirates; ‡ Center for Catalysis and Separation (CeCaS), Khalifa University, P.O. Box 127788, Abu Dhabi, United Arab Emirates; § Physics Department, Khalifa University, P.O. Box 127788, Abu Dhabi, United Arab Emirates; ∥ Department of Chemistry, Heterogeneous Catalysis Laboratory, University of Cyprus, 1 University Avenue, University Campus, 2109 Nicosia, Cyprus; ⊥ Oxide & Organic Nanomaterials for Energy & Environment (ONE) Laboratory, Advanced Membranes & Porous Materials (AMPM) Center, and KAUST Catalysis Center (KCC), Physical Science & Engineering (PSE), King Abdullah University of Science and Technology (KAUST), Thuwal 23955, Saudi Arabia; # Synchrotron-Light for Experimental Science and Applications in the Middle East (SESAME), Allan 19252, Jordan; ∇ Core Laboratories, King Abdullah University of Science and Technology (KAUST), Thuwal 23955-6900, Saudi Arabia

**Keywords:** high entropy oxides, ammonia, oxygen vacancies, isotopic exchange, DFT, EPR, Synchrotron EXAFS

## Abstract

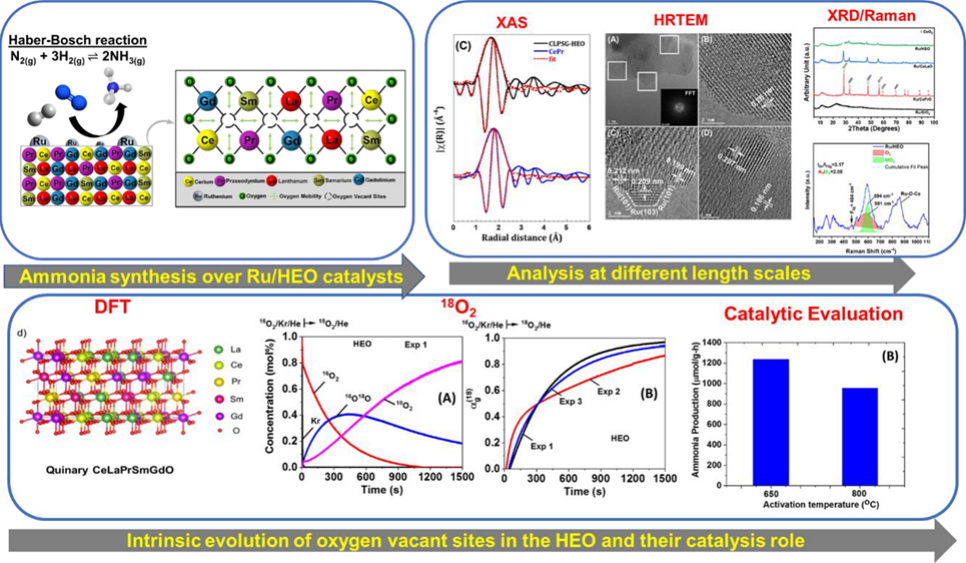

In this work, a relatively new class of materials, rare earth (RE) based high entropy oxides (HEO) are discussed in terms of the evolution of the oxygen vacant sites (O_v_) content in their structure as the composition changes from binary to HEO using both experimental and computational tools; the composition of HEO under focus is the CeLaPrSmGdO due to the importance of ceria-related (fluorite) materials to catalysis. To unveil key features of quinary HEO structure, ceria-based binary CePrO and CeLaO compositions as well as SiO_2_, the latter as representative nonreducible oxide, were used and compared as supports for Ru (6 wt % loading). The role of the O_v_ in the HEO is highlighted for the ammonia production with particular emphasis on the N_2_ dissociation step (N_2(ads)_ → N_ads_) over a HEO; the latter step is considered the rate controlling one in the ammonia production. Density functional theory (DFT) calculations and ^18^O_2_ transient isotopic experiments were used to probe the energy of formation, the population, and the easiness of formation for the O_v_ at 650 and 800 °C, whereas Synchrotron EXAFS, Raman, EPR, and XPS probed the Ce–O chemical environment at different length scales. In particular, it was found that the particular HEO composition eases the O_v_ formation in bulk, in medium (Raman), and in short (localized) order (EPR); more O_v_ population was found on the surface of the HEO compared to the binary reference oxide (CePrO). Additionally, HEO gives rise to smaller and less sharp faceted Ru particles, yet in stronger interaction with the HEO support and abundance of Ru–O–Ce entities (Raman and XPS). Ammonia production reaction at 400 °C and in the 10–50 bar range was performed over Ru/HEO, Ru/CePrO, Ru/CeLaO, and Ru/SiO_2_ catalysts; the Ru/HEO had superior performance at 10 bar compared to the rest of catalysts. The best performing Ru/HEO catalyst was activated under different temperatures (650 vs 800 °C) so to adjust the O_v_ population with the lower temperature maintaining better performance for the catalyst. DFT calculations showed that the HEO active site for N adsorption involves the O_v_ site adjacent to the adsorption event.

## 1 Introduction

Ammonia is maybe the most important chemical for humankind. The widely used, industrial-scale process for ammonia production is still dependent on the energy-demanding Haber–Bosch (HB) process (temperatures in the 723–773 K and pressures in the 15–30 MPa); the latter is consuming high amounts of energy (nearly above 1% of the global energy) in annual basis. Therefore, the design of processes that are more environmentally friendly and less energy-hungry is of pivotal importance.^1^ Shifting the energy demanding HB process to milder conditions would require a catalyst surface where N and H atoms are not bind strongly, while the activation barrier for the N_2_ to N dissociation is rather low; the latter is inversely dependent to the adsorbed atomic N stability (scaling relations).^2^

Fe and Ru are among the traditional catalysts for ammonia production with Fe being more acceptable for industrial use due to its lower costs.^3,4^ The dissociative mechanism involves the adsorption of N_2_ followed by its dissociation into 2N (atomic nitrogen) before its reaction with H_2_ and the production of NH_3(g)_. The energy-demanding step is that of N_2_ activation where the breaking of the N–N stable triple bond is involved; the latter is considered the rate-determining step on Ru and Fe widely used, catalysts. Therefore, a thorough understanding of the active sites and/or the catalyst design criteria involved in the N_2_ activation is crucial for optimizing the ammonia production rate.

Cerium dioxide (CeO_2_) is a widely used catalyst component for a span of applications from industrial and automobile exhausts^5^ to promote the water gas shift reaction,^6^ and hydrogen production reactions^7^ as well as solid electrolyte in fuel cells,^8^ due to its superior ability to recycle oxygen through redox reactions and high mobility of oxygen ion. The rapid formation and elimination of oxygen vacancy defects plays a crucial role in all these catalytic reactions. Ceria-based high-entropy oxides are expected to withhold great potential for catalytic reactions where the high oxygen mobility is a significant catalyst treatment,^9,10^ likely due to the theoretically predicted easiness of formation of the oxygen vacancies. Other properties of HEO that make them attractive candidates for catalysis/electrocatalysis are their high ionic/mixed conductivity due to their high lattice strain^11^ as well as the presence of multiple luminescent centers (origin of exceptional optical properties). In particular, the lattice distortion in the HEO contributes to properties such as good transparency, high refractive index, overall enhancing the imaging quality, and expanding viewing angle lens. Moreover, the stabilization of unconventional spin-electronic states can lead to noteworthy magnetic applications. It has been reported that the HEO with spinel structure responds quickly to small magnetic field changes. Additionally, the combination of morphological features with the tunability of the lattice distortion can give rise to distinct values of strength and elastic modulus (mechanical properties). Last but not least, the low thermal conductivity of the high entropy oxides is usually combined with fairly good electrical conductivity reaching the levels of steel and lead.^12−14^ In a recent review article by our group, the design criteria of the HEO are explicitly presented and discussed toward tuning the features that dictate their catalysis chemistry.^10^ As it has also been eloquently discussed in^15^ the multielemental nature of the HEO gives space for tunability of the active sites and/or vacancies location, whereas understanding the defects formation plays a major role in unveiling their functional properties and driving new discoveries. From a thermodynamic perspective, the driving force for defect formation is the configurational entropy increase, without undermining the role of the constituent elements.^9^

In the present study, the evolution of the formation of oxygen vacancy (O_v_) defects as a result of stepwise transition from pure ceria (single oxide), to binary, to ternary systems, and eventually to quinary rare earth high entropy oxide (HEO) systems is explored. The CeLaPrSmGdO composition was used as model one. Computational ab initio tools, as well as experimental techniques, such as Raman spectroscopy, X-ray photoelectron spectroscopy (XPS), electron paramagnetic resonance (EPR), and synchrotron X-ray absorption spectroscopy (XAS) along with detailed electron microscopy tools involving HRTEM, STEM-HAADF, RGB analysis, FFT analysis, and isotopic oxygen exchange transient experiments, are employed. Following the investigation of the O_v_ formation in the HEO composition, a demonstration of the role of O_v_ in the ammonia thermal production is provided over Ru based HEO supported catalyst with a critical comparison with Ru-based catalysts where the support is either binary reducible oxide (CePrO, CeLaO) or nonreducible one (SiO_2_). The impact of the support (HEO vs CePrO) on the growth of Ru particles shape and size is critically discussed whereas insights on the N_2_ dissociation over the Ru/HEO catalyst and the role of the O_v_ proximity to the N_2_ dissociation site (HEO active site) are provided.

## 2 Methods

### 2.1 Computational Studies

The first-principles calculations are based on density functional theory as this is developed in the Vienna ab initio Simulation package (VASP),^16,17^ using generalized gradient approximation (GGA) of Perdew, Burke, and Ernzerhof^18^ as the exchange-correlation functional. Projector-augmented-wave potentials are used to account for the valence electrons and ionic core interactions.^19,20^ 4f electrons of the rare-earth elements are considered core electrons for structural relaxations, while for computation of the total energy, 4f electrons are considered valence electrons. For computational efficacy, soft pseudopotentials of oxygen and nitrogen are used. The GGA+U method^21^ is used to push the unoccupied 4f orbitals of the rare-earth elements away from the Fermi level, and the Hubbard U parameter is set to 5 eV for all the rare-earth elements. Spin-polarized calculations are performed for structural relaxations, while for computations of the total energy spin–orbit interaction is taken into account. A fluorite-type crystal structure (with space group 225) of pure CeO_2_ is used for computations. The structure of the ternary and quinary high-entropy oxide (HEO) is generated using special quasirandom structures (SQS)^22^ based on a 144 and 180-atom supercell respectively on a parent CeO_2_ prototype structure, using alloy theoretic automated toolkit (ATAT) code.^23^ SQS has been successful in describing the electronic and thermodynamic properties of various disordered systems.^24^ Energy cutoff of 350 eV is used for plane wave basis set expansion and Γ-centered k-point mesh of 4 × 4 × 4, 3 × 5 × 2, 3 × 2 × 2, and 2 × 1 × 1 is used for pure CeO_2_, binary, ternary and quinary HEO, respectively. The oxygen vacancies on the (111) surface of these systems are considered, where a vacuum region of 20 Å is used in the supercell containing the surface to avoid spurious interactions with the periodic images. The formation energy (Δ*E*_form_) of oxygen vacancy is computed as follows:
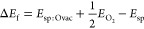
1where *E*_sp:Ovac_ is the energy of the supercell with one oxygen vacancy, *E*_O2_ is the energy of one oxygen molecule, and *E*_sp_ is the energy of the supercell without any oxygen vacancy.

#### 2.1.1 Ru on HEO Calculations

The literature revealed that the ruthenium cluster, having a pyramid structure composed of 4 atoms, supported on ceria is an energetically stable structure.^25^ Likewise, in this work, a similar Ru_4_ cluster was deposited atop the oxygen vacancy site on reduced quinary LaPrCeSmGdO10(111) surface (hereafter named the Ru_4_/HEO surface).

### 2.2 Experimental Studies

#### 2.2.1 Catalysts Preparation

##### 2.2.1.1 Metal Oxide Synthesis

In this study, the high entropy oxide (CeLaPrSmGdO) support and two reference (reducible) supports (i.e., CeLaO and CePrO) were synthesized via the coprecipitation method.^26^ The total moles of the metal nitrate precursors were maintained at 0.023 mol. For the CeLaPrSmGdO (HEO) support, the metal nitrate precursors with equimolar composition were dissolved in 45 mL DI water and placed on an orbital shaker with RPM set to 340 at 65 °C for 15 h. Then, 12.5 mL of ammonium hydroxide was added to the solution while stirring for an extra 2 h. The mixture was dried overnight at 100 °C, then calcined at 900 °C at a rate of 5 °C/min. A similar procedure was followed for the synthesis of reference supports, CeLaO and CePrO, except that the molar ratio of Ce to the metal (i.e., La and Pr) was 4:1. The SiO_2_ support was provided by Zeofree (commercial) and it was used as received.

##### 2.2.1.2 Ru-Based Catalyst Synthesis

Ruthenium nanoparticles were prepared by chemical reduction of RuCl_3_·xH_2_O using NaBH_4_ as a reducing agent and KOH as a stabilizer (in situ reduction). First, 50 mL of 2-propanol, 50 mL of DI water, and 360 mg of oxide support (HEO, CePrO, CeLaO, SiO_2_) were mixed together and sonicated for 20 min. Then, 0.047 g of RuCl_3_·*x*H_2_O was added to the mixture and sonicated for 5 more mins. The mixture was stirred on a high magnetic stirrer at 500 rpm for 10 min. Then, a mix of 0.1 M KOH and 0.1 M of NaBH_4_ (total 50 mL) was slowly added and stirred for an additional 1 h. The final mix was washed 3 times in DI water and then dried overnight at 90 °C. Therefore, the prepared Ru-based catalysts are the: Ru/HEO, Ru/CePrO, Ru/CeLaO, and Ru/SiO_2_ all with 6 wt % Ru loading.

#### 2.2.2 Characterization of Catalysts

Most of the techniques used in this study are described in previous studies.^27,28^ More details about Raman, XPS, EPR, and HRTEM are given in the SI.

##### 2.2.2.1 Synchrotron EXAFS

The X-ray absorption fine structure (XAFS) allows to assess the oxidation state by taking into account the X-ray absorption near edge structure (XANES) segment of the spectra; additionally, it helps to unveil structural characteristics at different length scales- short and medium range- through utilizing the extend X-ray absorption fine structure (EXAFS) part of data; the latter were collected on the BM08-XAFS/XRF beamline^29^ at Synchrotron-light for Experimental Science and Applications in the Middle East (SESAME), Jordan for all catalysts of this study following their reduction (10 vol % H_2_/Ar, 650 °C, 120 min). XAFS spectra were recorded, at room temperature, in step-by-step scanning mode at the Ce L_III_-edge (5723 eV). Transmission and fluorescence modes of the signal were collected in XAFS in both ionization chambers and the energy-selective single-element silicon drift detector (SDD) from KETEK GmbH, Germany. For scanning the energy, at the XANES region, with a step size of 0.2 and 0.5 eV for the pre-edge and white line, respectively, a double crystal monochromator equipped with Si(111) crystal was used. Variable energy steps using a fixed k space wavenumber of 0.03 A^–1^ were utilized for recording the EXAFS region of the spectra. Demeter^30^ and WinXAS^31^ software packages were used for the collection and processing of the XAFS data. The reference spectra used for XANES are from the databases^32,33^ for the CeO_2_, CePO_4_, and CeF_4_.

##### 2.2.2.2 ^16^O/^18^O Surface Isotopic Exchange

The solid in powder form (50 mg, dp <106 μm) was introduced into a quartz CSTR microreactor,^34^ where the absence of interparticle (external) and intraparticle (internal) concentration gradients was verified.^35^ Both catalysts were pretreated as follows: (i) Exp 1: Calcination (5% O_2_/He) at 600 °C/1 h; (ii) Exp 2: Calcination (5% O_2_/He) at 600 °C for 1 h, Reduction (5% H_2_/He) at 600 °C/2 h; (iii) Exp 3: Calcination (5% O_2_/He) at 600 °C for 1 h, Reduction (5% H_2_/He) at 800 °C/2 h. Transient Isothermal Oxidation (TIO) was used to investigate the formation of oxygen vacancies as a function of the pretreatment procedure over the two solids. More specifically, following the catalyst pretreatment, the reactor was purged in He for 30 min at 600 °C, followed by a step-gas switch to 1% ^16^O_2_/1% Kr/Ar/He (F_T_ = 100 N mL min^–1^). It should be mentioned that at the end of TIO, steady-state rates of oxygen transfer between gas-phase oxygen and lattice oxygen (O_L_)/oxygen vacancies (O_v_) of the solid are established. TIO experiment was followed by a transient isothermal isotopic exchange with ^18^O_2_ experiment (^18^O_2_-TIIE) in order to probe the kinetics of ^16^O/^18^O surface isotopic exchange and bulk oxygen diffusion (oxygen mobility) under equilibrium or pseudo equilibrium conditions,^36^ verified by the sum (1 mol %) of all three isotopologues oxygen gas compositions during the whole transient. It is worth mentioning that the shape and the position of the ^16^O^18^O species strongly depend on the bulk oxygen diffusion coefficient (*D*_eff_, cm^2^ s^–1^) of the solid, but also the amount of oxygen (N^16^O, mmol g^–1^) available to be exchanged.^37^ Specifically, during ^18^O_2_-TIIE the following gas step switch was applied: 1% ^16^O_2_/1% Kr/Ar/He → 1% ^18^O_2_/Ar/He at 600 °C. During the ^18^O gas mixture, the transient responses of ^16^O_2_, ^16^O^18^O, ^18^O_2_, and Kr (*m*/*z* = 32, 34, 36, and 84, respectively) were continuously monitored via an online mass spectrometer (Balzers, Quadrupole 1–200 amu), and converted into concentration (mol %) by using certified gas mixtures (i.e., 2% ^16^O_2_/He, 5% ^18^O_2_/He, and the impurity concentration of ^16^O^18^O(g) present in the 5% ^18^O_2_/ He).

The N^16^O (mmol g^–1^) amount of ^16^O/^18^O exchanged (oxygen storage capacity, OSC) was estimated via the material balance depicted in eq 2:
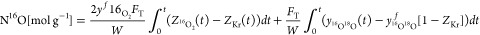
2where *Z_i_* and *y_i_* are the dimensionless response and mole fraction of gaseous species *i*, respectively.

The dimensionless  descriptor function illustrates both the surface ^16^O/^18^O exchange (initial period under ^18^O gas), which is crucial from an industrial point of view, and the oxygen diffusion in the bulk (prolonged time on ^18^O gas), as obtained via eq 3. It should be noted that the lower the value of , the faster the oxygen diffusion in the bulk of the solid.
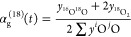
3

#### 2.2.3 Catalytic Evaluation

Catalytic ammonia synthesis tests were conducted within an Avantium system (Flowrence XD, Netherlands) using a quartz tube reactor with an I.D. of 2 mm and an O.D. of 3 mm, equipped with a porosity 3 filter. Four reactors were simultaneously employed for screening purposes, with each reactor loaded with 25 mg of catalyst, which was diluted with 600 μL of silicon carbide (SiC) grit (Lot: 10226827, Alfa Aesar). One reactor was kept as a blank control, containing only SiC. Before the activity measurement, the catalysts were treated in an in situ reduction/activation process. This was achieved under a gas mixture consisting of hydrogen (99.999%) and nitrogen (99.999%) at a 3:1 ratio, with a total flow rate of 15 mL min^–1^ at 650 °C (ramping rate: 5 °C min^–1^) for 2 h. Catalytic performance assessments were conducted at 10 bar, 400 °C. For these experiments, precise gas flow rates of 11.25 for hydrogen and 3.75 mL min^–1^ for nitrogen (corresponding to a weight hourly space velocity of 36,000 mL g_cat_^–1^ h^–1^) were maintained using mass flow controllers. An online gas chromatogram (8890 GC system, Agilent) was employed to analyze the reaction products under isothermal conditions. Helium (99.999%) at a flow rate of 1.25 mL min^–1^ was used as an internal standard for quantitative analysis. All experiments were replicated three times, and the average values were utilized for generating graphical representations and data analysis. To eliminate the scenario of spurious NH_3_ presence (e.g., due to possible impurities in the lines), control experiments were performed following the procedure: the same experimental protocol (reduction at 75% H_2_ −25% N_2_ at 650 °C for 2 h) followed by the reaction conducted in the absence of N_2_ in the feed (control experiment), where gas flow of H_2_ 15 mL/min, and He 1.25 mL/min, at 400 °C, and pressure of 10 bar was used.

## 3 Results and Discussion

### 3.1 Computational Studies

#### 3.1.1 Structural Stability

**Figure 1:**
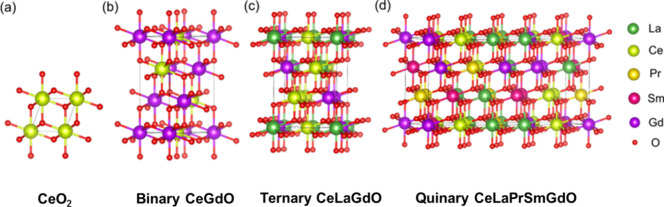
Unit cells of (a) pure CeO_2_, (b) binary CeGdO, (c) ternary CeLaGdO, and (d) quinary CeLaPrSmGdO.

Ceria exists in fluorite cubic crystal structure with space group no. 225 as shown in Figure 1a. The formation energy of ceria is −4.04 eV/atom (shown in Table 1). To understand the formation of oxygen vacancy in high-entropy systems calculations for Ce-based binary systems, CeGdO_4_ (coded as CeGdO), CePrO_4_ (coded as CePrO), ternary systems, CeLaGdO_6_, CeLaPrO_6_, (coded as CeLaGdO and CeLaPrO) and quinary high-entropy oxide CeLaPrSmGdO_10_ (coded as CeLaPrSmGdO) were performed. The crystal structure of the binary system is derived from the ordered arrangement of rare earth atoms in the crystal structure of ceria. For ternary and quinary high entropy systems, crystal structure is derived by SQS^22^ using the ATAT package.^23^ The bulk crystal structures for pure CeO_2_, binary CeGdO, ternary CeLaGdO, and quinary CeLaPrSmGdO are shown in Figure 1. The formation energy for binary, ternary, and quinary bulk phases is mentioned in Table 1. It is to be noted that the formation energy is negative even for high-entropy systems, suggesting that although configurational entropy contributes to the stability of these systems, it is not the governing criterion for stability, as these materials are stable even at *T* = 0 K.

**Table 1:** Formation Energy in eV/fu for CeO_2_ and CeO_2_-Based High Entropy Systems

	Δ*H*_f_ (eV/atom)
CeO_2_	–4.04
binary (CeGdO)	–4.83
binary (CePrO)	–3.76
ternary (CeLaGdO)	–4.26
ternary (CeLaPrO)	–3.54
quinary (CeLaPrSmGdO)	–4.13

#### 3.1.2 Evolution of Oxygen Vacant Sites

**Figure 2:**
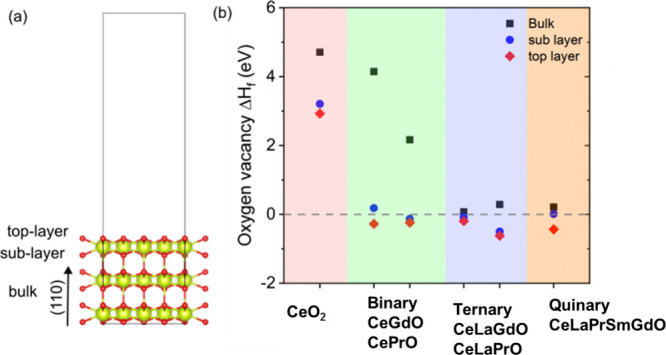
(a) Unit cell of CeO_2_ along (110) surface with vacuum. Bulk region, sublayer, and top-layers are shown. (b) Evolution of oxygen vacancy formation energy in pure CeO_2_, binary systems (CeGdO, CePrO), ternary systems (CeLaGdO, CeLaPrO), and quinary (CeLaPrSmGdO) high entropy system.

To understand the evolution of oxygen vacancy formation in ceria-based high entropy systems, the oxygen vacancy formation energy (Δ*E*_f_) along (111) surface for each of the cases, pure CeO_2_, binary, ternary, and quinary systems was computed. As shown in Figure 2a the formation energy of oxygen vacancy defect using eq 1 in bulk, sublayer, and top-layer along (111) surface of various systems was computed. For pure CeO_2_ the Δ*E*_f_ in bulk is 4.71 eV, on the top-layer is 2.93 eV and the sublayer is 3.21 eV, which is in agreement with previous studies.^38,39^ Similarly, Δ*E*_f_ is computed for different systems (shown in Figure 1) along (111) surface, values shown in Table 2 and graphically shown in Figure 2b.

**Table 2:** Formation Energy (Δ*H*_f_) in Top-Layer, Sub-Layer, and Bulk for Various Systems

		Δ*E*_f_ (eV)
CeO_2_	top-layer	2.93
	sublayer	3.21
	bulk	4.71
binary (CeGdO)	top-layer	–0.28
	sublayer	0.18
	bulk	4.15
binary (CePrO)	top-layer	–0.24
	sublayer	–0.13
	bulk	2.17
ternary (CeLaGdO)	top-layer	–0.20
	sublayer	–0.09
	bulk	0.07
ternary (CeLaPrO)	top-layer	–0.62
	sublayer	–0.50
	bulk	0.29
quinary	top-layer	–0.43
(CeLaPrSmGdO)	sublayer	0.01
	bulk	0.21

Several possible sites of oxygen vacancy in bulk and on (111) surface are considered and the one with the lowest energy is considered for further analysis. It is observed that as the number of heteroatoms increases, moving from pure ceria to mixed cation systems, Δ*E*_f_ reduces and eventually reaches values below zero, suggesting that in high-entropy systems (HEO) oxygen vacancy (O_v_) formation is energetically more favorable as compared to pure CeO_2_. This is due to the ease of transition of cation from +4 oxidation state to +3 oxidation state. Pr, Sm, and Gd exhibit various oxidation states, including +3 and +4. + 3 oxidation state of La, Pr, and Gd is more favorable as they exhibit fully filled [Xe], [Xe] 6s^2^, and [Xe] 4f^7^ electron configuration. Therefore, replacing Ce with these rare-earth elements *tends to ease the formation of oxygen vacancy both in bulk and on the surface*. It is also noticeable that Δ*E*_f_ is highest in bulk, and gradually reduces in sublayer, and is lowest at the surface. This trend is observed in all the systems. This can be explained by the fact that the oxygen atoms on the (111) surface of these ceria-based systems contain dangling bonds, such that the surface is electron-poor. Removal of an oxygen atom on the surface provides extra charge from the cations which are near to the vacancy site, to the already electron-poor surface, thereby favoring the formation of oxygen vacancy.

### 3.2 Experimental Studies: Monitoring of the HEO vs Binary Oxides Differences

Further understanding of the fundamental differences of the HEO, compared to (1) simpler structures of ceria-based oxides (e.g., binary supports, such as CeLaO, CePrO) and (2) nonreducible ones (Silica) was herein attempted using a toolkit of analytical techniques. For this reason, the catalysts with Ru/CeLaO, Ru/CePrO, Ru/SiO_2,_ and Ru/HEO compositions were investigated; more details are provided in what follows.

#### 3.2.1 Overview of Structural/Textural and Morphological Features

**Figure 3:**
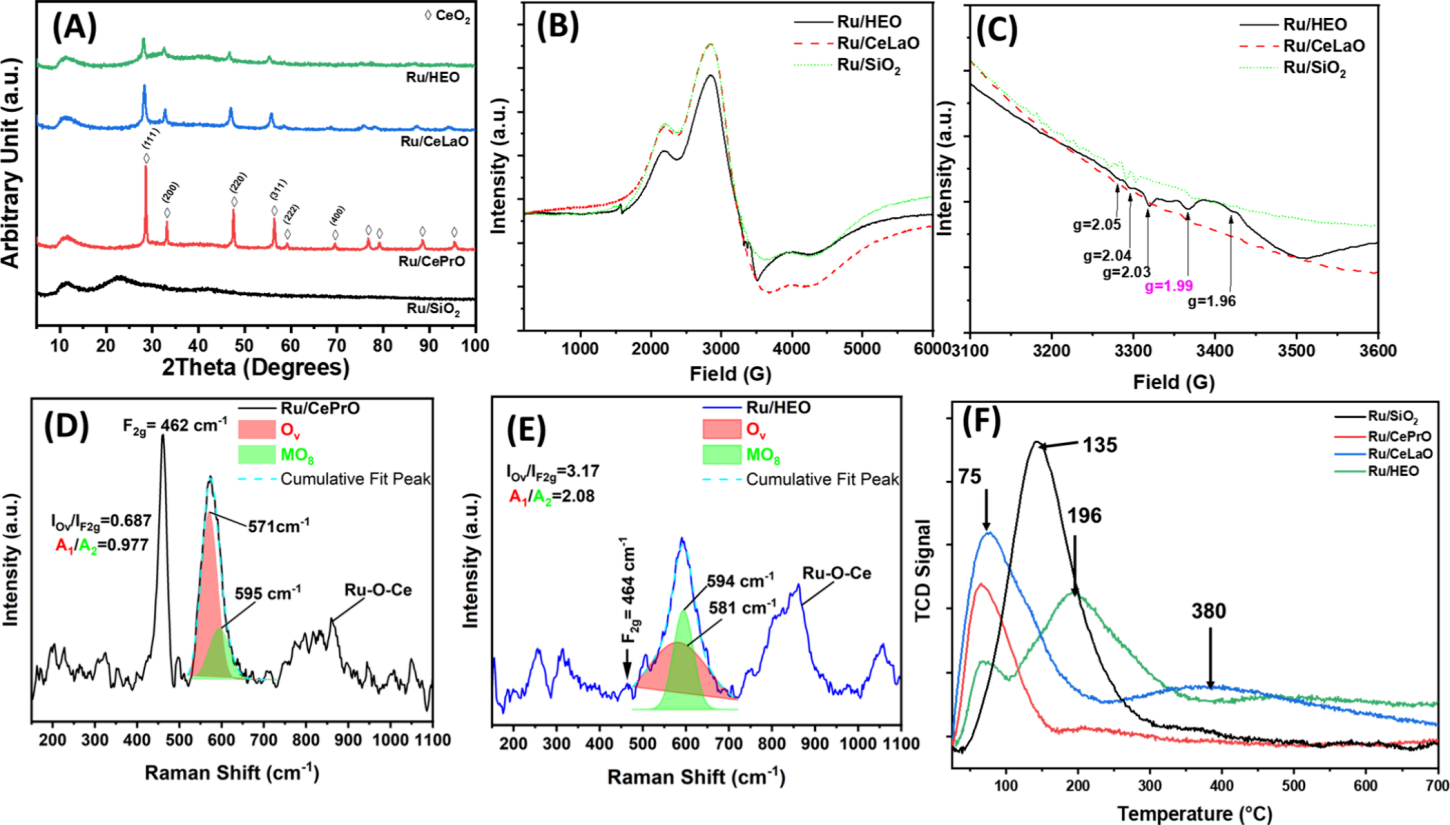
(A) XRD diffractograms; (B–C) EPR spectra collected at 100 K; (D) Raman spectra collected over the Ru/CePrO catalyst; (E) Raman spectra collected over the Ru/HEO catalyst; (F) H_2_-temperature-programmed desorption (H_2_-TPD) profiles obtained over all the Ru catalysts of the present study.

Figure 3A presents the XRD patterns of the Ru-based catalysts on different supports of this study following their reduction at 650 °C for 2 h under an H_2_ atmosphere; the supports were CePrO, CeLaO, HEO, and silica. All the ceria-based supports present the characteristic reflections of the fluorite ceria lattice; namely 33.2°, 47.6°, 56.5°, 59.1°, 69.4°, 76.6°, and 79.2° are assigned to the diffractions of (111), (200), (220), (311), (222), (400), (311), and (420) crystallographic planes of CeO_2_.^40^ The catalyst where silica was used as support presents a rather amorphous pattern, whereas the Ru/HEO catalyst presents no reflection due to a dopant relevant phase (e.g., La_2_O_3_, Sm_2_O_3_, Gd_2_O_3_, Pr_2_O_3_, or combination/solid solution of them). On the other hand, despite the relatively high Ru loading (6 wt %), there is no obvious reflection peak corresponding to Ru/RuO_2_ phases, demonstrating the high dispersion of Ru and/or RuO_2_ phases in all the supports herein used (regardless of their nature: reducible or nonreducible). N_2_ adsorption at 77 K (Figure S1) over all the ceria-based supported catalysts showed similar BET surface areas (Table S1) of the catalysts Ru/CeLaO (18 m^2^/g), Ru/CePrO (6 m^2^/g), and Ru/HEO (18 m^2^/g), whereas the pore volume was found to be in the 0.032–0.048 cm^3^/g range for the ceria-based catalysts and 0.936 cm^3^/g for the silica-based catalyst. The Ru/SiO_2_ catalyst had a surface area of 102 m^2^/g. All the catalysts presented similar morphological features (rather aggregates formation based on the SEM studies with the Ru/SiO_2_ catalyst presenting a bit more spongy morphology (Figure S2). The latter is in agreement with the high surface area of this catalyst. Elemental EDS mapping measurements, by SEM, were carried out to confirm the presence of elements, and the results are presented in Table S2. Based on the values reported, the EDX-derived values for the Ce/La and Ce/Pr ratios are close to the nominal one (4/1); the same applies to the Ce/La/Sm/Gd/Pr ratio which reflects the equimolar composition of the HEO (design criterion is fulfilled).

#### 3.2.2 Probing the Oxygen Vacant Sites and the Metal–Support Interactions

Figures 3B,C and S3 present the EPR spectra of the Ru/CeLaO (reducible oxide, reference), Ru/HEO, and Ru/SiO_2_ (nonreducible, reference) obtained at 100 K and room temperature, respectively, following their reduction at 650 °C for 2h in H_2_ atmosphere. EPR is used in this study as a diagnostic tool for the Ce^3+^ species; given their paramagnetic nature, Ce^3+^ ions can be detectable using this technique. From the catalysis perspective, it is of high importance to be able to probe the presence of oxygen vacancies (O_v_); the formation of the latter is associated with the Ce^3+^ presence. It is worthwhile to discuss in brief the mechanisms of O_v_ formation in these materials; according to the first mechanistic scenario, as the lattice oxygen leaves its original position, the two electrons in the vacant site localize on two Ce^4+^ ions and they reduce them forming Ce^3+^–V_O_–Ce^3+^ pairs.^41^ Usually, conventional EPR at RT (298 K) cannot detect these species due to the strong spin–spin interaction. An alternative mechanism of oxygen vacancy (O_v_) formation is when one electron, originating from lattice oxygen, localizes on a Ce^4+^ leading to the formation of Ce^4+^–V_O_–Ce^3+^ species; in this scenario, the other electron is trapped in an adjacent to the vacancy position, leading to the formation of F+ center;^41^ the latter can be probed by RT (298 K) EPR. In the open literature, there is a lot of discussion on the origin of peak with *g*_av_ ∼ 1.97 which is usually assigned to Ce^3+^. Based on the literature, the symmetry around the Ce^3+^ ions dictates the *g* parameter value. As Ce^4+^ to Ce^3+^ reduction takes place, it leads to the formation of oxygen vacancies accompanied by lattice distortion. In the case of CaF_2_ (isomorphic) for Ce^3+^ ions in a coordination environment of low symmetry, *g* ∼ 3.67 has been reported, whereas lines with *g* values of 2.4 for the trigonal site of Ce^3+^ ions have also been reported.^41^ As can be seen in Figure 3C, EPR signals at different *g*-values confirm the various paramagnetic centers; of high significance is the signal at *g* ∼2.000 which is assigned to superoxide anions (•O^2–^) attached to Ce^4+^ ions; the intensity of this pulse is correlated with the concentration of oxygen vacant sites.^42^ This signal is of high intensity in the case of the Ru/HEO catalyst.

Additional structural information was acquired utilizing Raman spectroscopy over the Ru-based catalysts of this study following their reduction at 650 °C for 2h in H_2_ atmosphere. In particular, Raman spectroscopic results (Figure 3D,E) obtained over the Ru/CePrO and Ru/HEO catalysts give insight into the effect of dopants on the multi-elemental HEO structure and defective sites formation as well as how the two different supports interact with the metal sites (Ru) through strong metal support interactions (SMSI). A comparative Raman plot is given in Figure S4 to assist with the peaks’ assignment. It is known that the Raman spectra for typical ceria-based materials have a dominant peak, the F_2g_ peak, usually found at about 464 cm^–1^ and assigned to the symmetric stretch mode of the Ce–O_8_ crystal unit, characteristics of the fluorite lattice structure.^43^ A close look at the Raman spectra of the catalysts (Figure S4) reveals significant changes in them indicating the remarkable impact of the dopants in the ceria microstructure owing to higher defectiveness and a rise in topographical/local disorder. Particularly, the F_2g_ peak appears quite sharp in the case of Ru/CePrO catalyst and rather suppressed in the case of Ru/HEO catalyst (see Figures 3E and S4); this is associated with the high degree of oxygen sublattice distortion in the latter case (HEO) and the high sensitivity of Raman spectroscopy to capture the oxygen sublattice disturbances. The herein Raman spectra over the HEO agree quite well with the literature reported by Sarkar et al.^44^ Apart from the suppressed intensity of the F_2g_ peak, changes in the defect-induced bands were found in the 500 to 700 cm^–1^ range, which was being misinterpreted in the open literature as oxygen vacancies associated with the presence of reduced Ce^3+^^45−47^ species or to oxygen vacancies (O_v_) involving movement of an oxygen atom into an octahedral interstitial position to obtain vacancy.^48,49^ The characteristic features have rightly been considered intrinsic properties of a pure ceria structure; however, intensity and broadness of the defects band are traced to extrinsic defects, attributed to the addition of dopant (Gd, Sm, La, and Pr for HEO; Pr for CePrO).^50^ The abundant defects as those identified in the Raman spectra are linked to the strong interaction between ceria and doped elements, thereby obtaining a microstructural change. The introduction of dopant into the ceria microstructure causes a steady increase in the amount of intrinsic and extrinsic defects. In a more quantitative manner, this is reflected in the value of the I_Ov_/I_F2g_ ratio, the latter being a descriptor of the O_v_ abundance. In particular, the I_Ov_/IF_2g_ ratio equals 0.68 and 3.2 in the cases of Ru/CePrO and Ru/HEO catalysts, respectively, demonstrating the abundance of O_v_ in the HEO lattice (bulk) and in the medium range that is probed by Raman scattering effects.^51^ Additionally, the Raman band at 1100 cm^–1^ corresponding to the Ru–O–Ce bond environment demonstrates strong metal–support interactions (SMSI); this band is sharp (higher signal-to-noise ratio) in the case of quinary HEO-based catalyst compared to the binary CePrO-supported one. The presence of Ru–O–Ce species is a critical factor for the Ru dispersion, as this has been assessed through H_2_ chemisorption studies (Figure 3F), and catalytic activity as it will be discussed later.^52,53^

#### 3.2.3 Surface Composition and Coordination Environment

**Figure 4:**
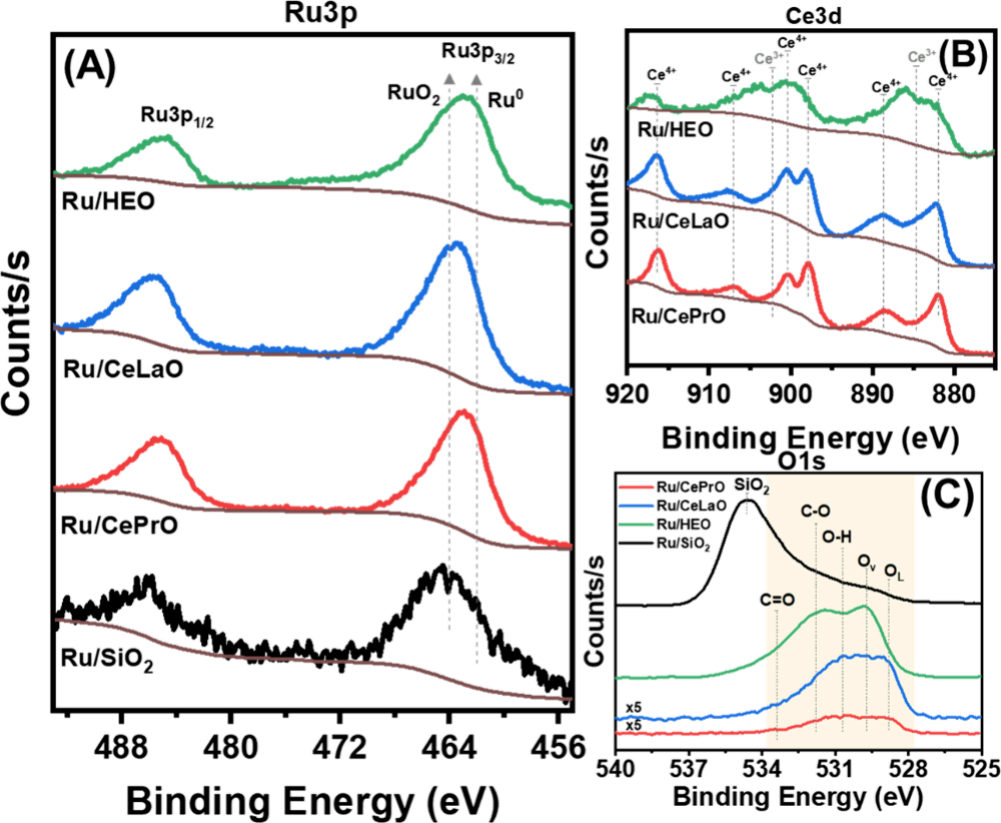
(A) Ru 3p, (B) Ce 3d, and (C) O 1s XPS core-level spectra of the prepared catalysts.

To delve deeper into the chemical states of Ru species in the as-prepared catalysts, X-ray photoelectron spectroscopy (XPS) measurements were conducted (see Figure 4A). Of particular interest in the XPS studies was to shed light on the effectiveness of the in situ Ru reduction as well as on the Ru-support interaction while Ru growth and reduction simultaneously take place during the synthesis. The peaks observed at 461.9 and 484.1 eV are attributed to metallic Ru, while those at 463.7 and 485.9 eV are associated with RuO_2_, as reported by Wang et al.^54^ A shift to lower binding energy in the Ru 3p core level spectra, particularly when compared to the Ru 3p of the reference Ru/SiO_2_ catalyst suggests changes in the electronic structure or chemical environment of Ru in the ceria-supported catalysts. The surface analysis reveals an increase in Ru/Ce ratio (Ru surface enrichment) as we transition from the binary system (i.e., Ru/CePrO or Ru/CeLaO) to the high-entropy system (Ru/HEO), as shown in Table 3. This observation suggests a different interaction among the three under study supports (CePrO, CeLaO, HEO) and the Ru species, promoting a high dispersion of Ru on the surface (in the case of HEO); these results are consistent with H_2_-TPD (chemisorption) results and the existing literature.^55^ Possible parameters contributing to the support-metal interaction are surface termination (dangling bonds, functional groups) and surface energy (J/m^2^). The Ru 3p spectra of Ru/CePrO, Ru/CeLaO, and Ru/HEO catalysts were fitted by CasaXPS software using Lorentzian Asymmetric line shape with Voigt function, and results are shown in Figure S5. Indeed, Ru species exist as Ru^0^ and Ru^4+^ (in RuO_2_), with the latter species being more dominant on the surface of the Ru/CeLaO supported catalysts (Ru^4+^/Ru^0^=15.6), as can be seen in Table 3. The ratios of Ru^4+^/Ru^0^ in Ru/CePrO, Ru/CeLaO, and Ru/HEO were found to be 5.5, 15.6, and 6.2, respectively. The increased presence of Ru^4+^ species in the Ru/CeLaO system, as indicated by the higher Ru^4+^/Ru^0^ ratio, suggests that the specific combination of dopants in the CeLaO system has a pronounced effect on the reducibility of Ru due to the strong metal–support interactions (SMSI).

**Table 3:** XPS Data of the Prepared Catalysts

catalyst	O (at %)	Ru (at %)	Ce (at %)	Ru/Ce	Ru^4+^/Ru^0^^a^
Ru/SiO_2_	50.53	0.37			
Ru/CePrO	13.01	24.31	9.82	2.5	5.5
Ru/CeLaO	22.70	7.97	7.78	1.0	15.6
Ru/HEO	26.58	3.48	0.9	3.9	6.2

a Values were estimated by XPS peak fitting using CasaXPS software.

The Ce 3d spectra depicted in Figure 4B are intricate, featuring multiple peaks that are labeled based on previous XPS studies on cerium oxide.^55,56^ The prominent peaks appearing at 916.2, 907, 900.3, 897.8, 888.7, and 882 eV are ascribed to Ce^4+^ species, whereas the two peaks observed at 902.2 and 884.6 eV are contributions of Ce^3+^ species. With respect to the binary systems, the Ru/HEO catalyst exhibits a shift of binding energy in higher values indicating a change in the Ce environment upon doping. Overall, a remarkable decrease in surface Ce species is observed (see Table 3), as well as intensity increase in Ce^3+^-related peaks can be visualized based on its spectrum in Figure 4B, likely attributed to the formation of extrinsic defects like oxygen vacancies due to doping with multiple elements.

In the O 1s spectra (Figures 4C and S5), the peak at approximately ∼528.8 eV is attributed to lattice oxygen, while the peaks around ∼530 eV, 531, and 533 eV are linked to oxygen vacancies, surface hydroxyl species, and carbonates, respectively.^57^ A significant change in the O environment is noticed across the catalysts. Moving from binary systems (e.g., CePrO and CeLaO) to the HEO system reveals increased peak intensities. This indicates that varying the chemical compositions has an impact on the type and concentration of surface oxygen species due to the formation of distinct metal oxides or mixed-metal oxides. Ru/HEO presents the highest content of surface oxygen species, likely attributed to the optimal formation of Ru–O–Ce bonds. Deconvolution of the O 1s peak (Figure S6 and Table S3), although, revealed that for the Ru/CePrO, Ru/CeLaO, and Ru/HEO catalysts the lattice oxygen (O_L_) abundance on the surface is 20, 28, and 42%, respectively. This result leads to the conclusion that the Ru/HEO catalyst has the least O_v_ surface concentration. Caution should be exercised in the interpretation of this data due to the fact that these values are only a rough estimation and will probably not give the correct stoichiometry, which presumably is (Ce, X, Y, Z)O_2_ in the case of HEO. Furthermore, the higher abundance of O_lattice_ (associated with lower abundance of O_v_) in the case of HEO catalyst can be understood by taking into consideration the following: (1) the XPS studies were performed on the as-prepared catalyst; (2) the XPS experiment was of ex-situ nature; (3) the fact that O_v_ creation can happen under the vacuum-catalyst interaction (ultrahigh vacuum in the XPS experiment) and (4) XPS experiment captures only the surface, so O_v_ at the different locations (subsurface/bulk) escape the detection depth of XPS. Additionally, the dynamics of O_v_ is a parameter to not be ignored. As pointed out by Younis et al.^58^ at high levels of doping (>20%) the vacancies become immobile due to their clustering and this can lead to deterioration of catalytic activity, as it will be discussed later.

#### 3.2.4 Coordination Environment through Synchrotron EXAFS Studies

Long and medium-range features, as those can be derived using XRD (long-range) and Raman (medium), can be hardly used to describe structural differences for materials in the nm-range. Local distortion with no periodicity can dictate the local structure and the ultimate functionality of a material.^59^ Synchrotron EXAFS was used to study the Ce–O correlations in a changing environment from the binary Ce–Pr oxide to the HEO.^60^

**Figure 5:**
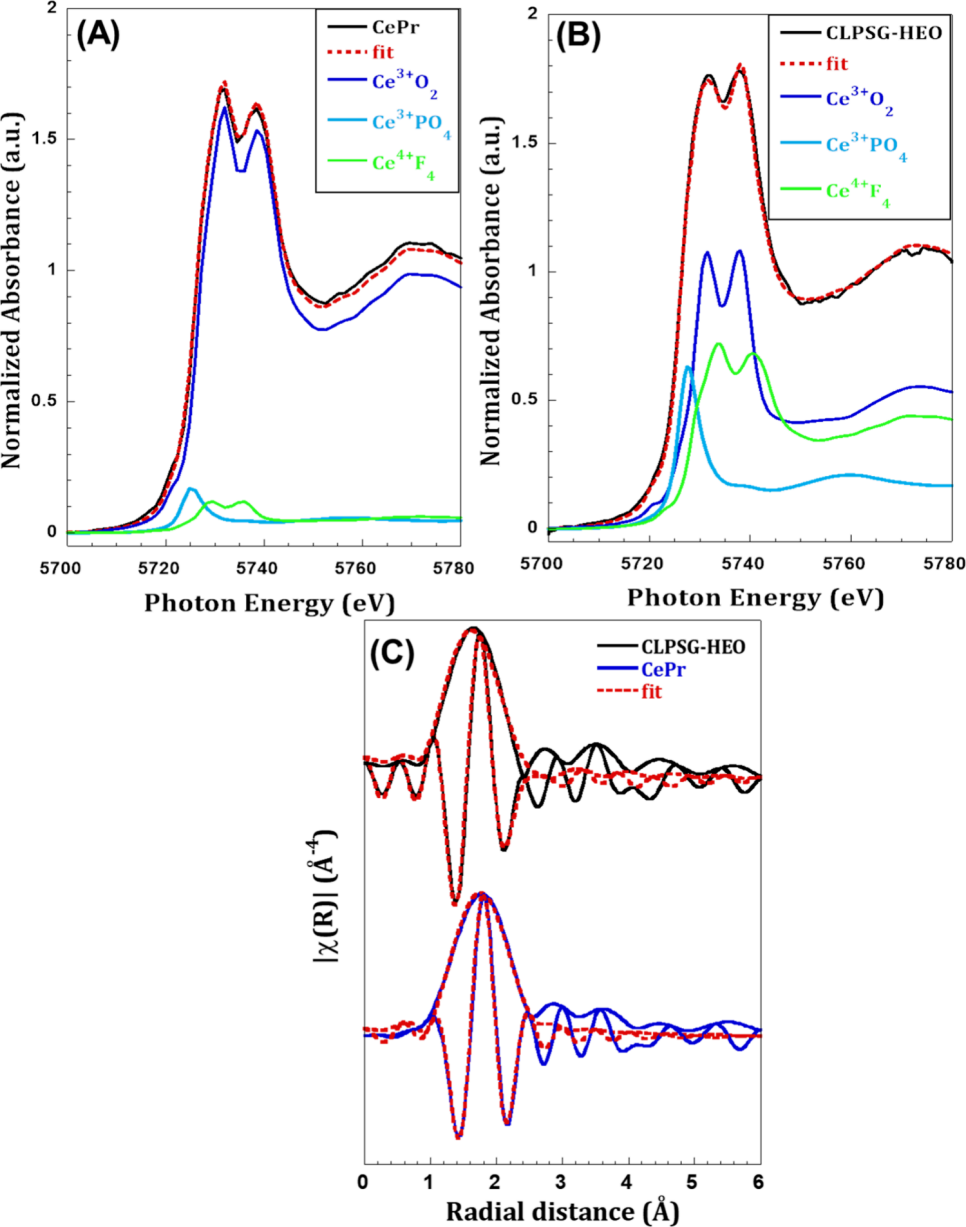
(A, B) Linear combination fitting of the XANES spectra collected at the LIII edge of Ce in the HEO sample (left) and CePrO sample (right) to determine all the oxidation states of Ce in the samples; (C) Fourier transforms with their respective real part of the k3 weighted EXAFS spectra collected at the Ce LIII edge in CLPSG-HEO and CePr samples.

The linear combination fitting (Figure 5A,B) of the **XANES** data collected at the L3 edge of Ce reveals a mixture of Ce^3+^ and Ce^4+^ oxidation states. In particular, the dominating oxidation state in the Ru/CePrO catalyst is Ce^3+^ at an abundance of ∼96%; in the case of the Ru/HEO catalyst, the Ce^3+^/Ce^4+^ ratio is about 1.2. The above results are important, though should be interpreted given the short-range sensitivity of the technique (different length scale with the focus being primarily around the atoms of interest).

Analyzing a **short EXAFS** signal can give good information on the first coordination shell around Ce (nearest neighbor, NN). Derived structural parameters from the EXAFS fitting show a coordination number of about 7 oxygen atoms with an interatomic distance that is close to that of CeO_2_ with a slight contraction in CePr to 2.30 Å and even shorter in the case of the HEO sample. In particular, the Ce–O distance becomes smaller as we move from ceria (2.34 Å) to CePrO (2.30 Å) to HEO (2.27 Å) (Table 4). The short distance coincides also with a reduction of the coordination number from 8 oxygen atoms for CeO_2_ to an average number of 7.4 and 7.1 atoms in CePr and HEO samples, respectively. These results may suggest a defect in the local structure at short-medium (could be defects or distortion). It should be mentioned here that, it was not possible to collect **long EXAFS** data (signal) at the L3 edge of Ce (due to the presence of the L2 edge at around 400 eV), and thus the coordination number is largely affected by the noise level which is reflected in the higher standard deviation (2.4 for N and 0.036 for the R, Table 4). This can be considered as a limitation of the above data, making EXAFS results only complementary to the above XANES ones. Moreover, based on the above HEO seems to be evolving as a triclinic CeO_2_ which has a shorter Ce–O distance (2.27) than the CeO_2_ cubic (2.34).

**Table 4:** Structural Parameters Derived by EXAFS Fitting of the First Shell around the Ce Atom in CLPSG-HEO and CePr Samples

sample	bond	*N* (atom)	*R* (Å)	σ2 (Å^2^)	Δ*E*(eV)
HEO	Ce–O	7.1 ± 2.4	2.27 ± 0.036	0.01460 ± 0.004	3.99 ± 1.6
CePrO	Ce–O	7.4 ± 2.0	2.30 ± 0.010	0.01542 ± 0.001	4.05 ± 0.5
CeO_2_ (cubic)	Ce–O	8^a^	2.34248^a^		

a Theoretical value.

#### 3.2.5 Ru Particle Shape and Size on the Different Supports (Electron Microscopy and Chemisorption Studies)

In the present study, the Ru dispersion and particle size were evaluated using H_2_ chemisorption studies, where the H atoms dissociative adsorb onto the Ru metal with a H:Ru stoichiometry of 1:1. Additionally, HRTEM analysis was employed to verify the chemisorption studies. Figure 3F displays the H_2_-TPD profiles of the Ru-based catalysts of this study. According to the open literature, the peak at low temperature (75 °C, 135 °C) is assigned to the desorption of hydrogen from various active sites (metallic Ru of different sizes), whereas the peak with *T*_max_ > 380 °C corresponds to the spillover hydrogen from the support. The corresponding Ru particle size was found to be in the 4–11 nm range demonstrating different extents of Ru dispersion as shown in Table S4. This Ru particle size variation, as probed using chemisorption, is expected to have a profound impact on the NH_3_ synthesis catalytic activity due to the well-known structural and size sensitivity of the reaction at hand and particularly of the N_2_ activation step.^61^

**Figure 6:**
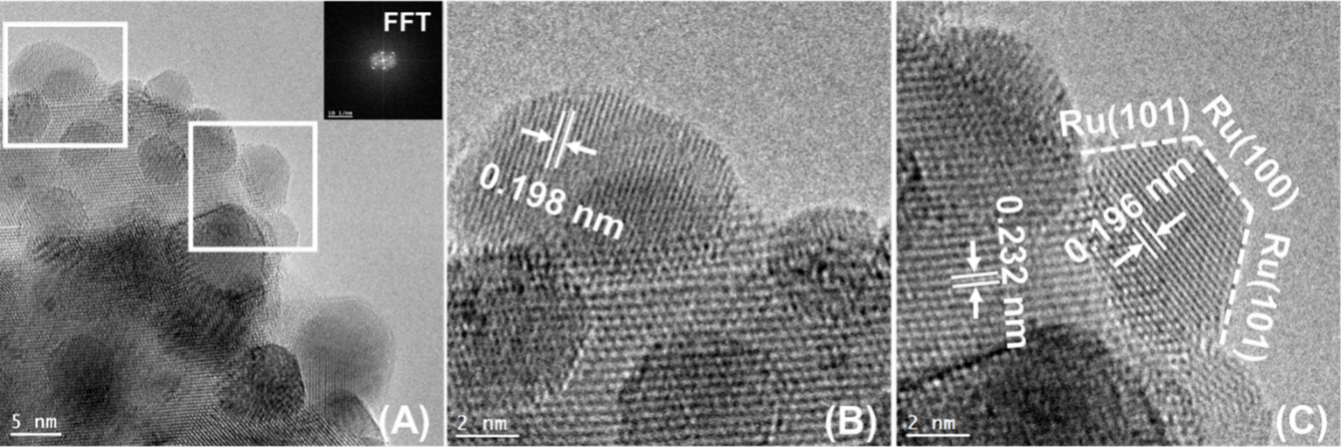
(A) HRTEM images along with Fast Fourier Transform (FFT) pattern; (B, C) facets analysis of the Ru/CePrO catalyst.

**Figure 7:**
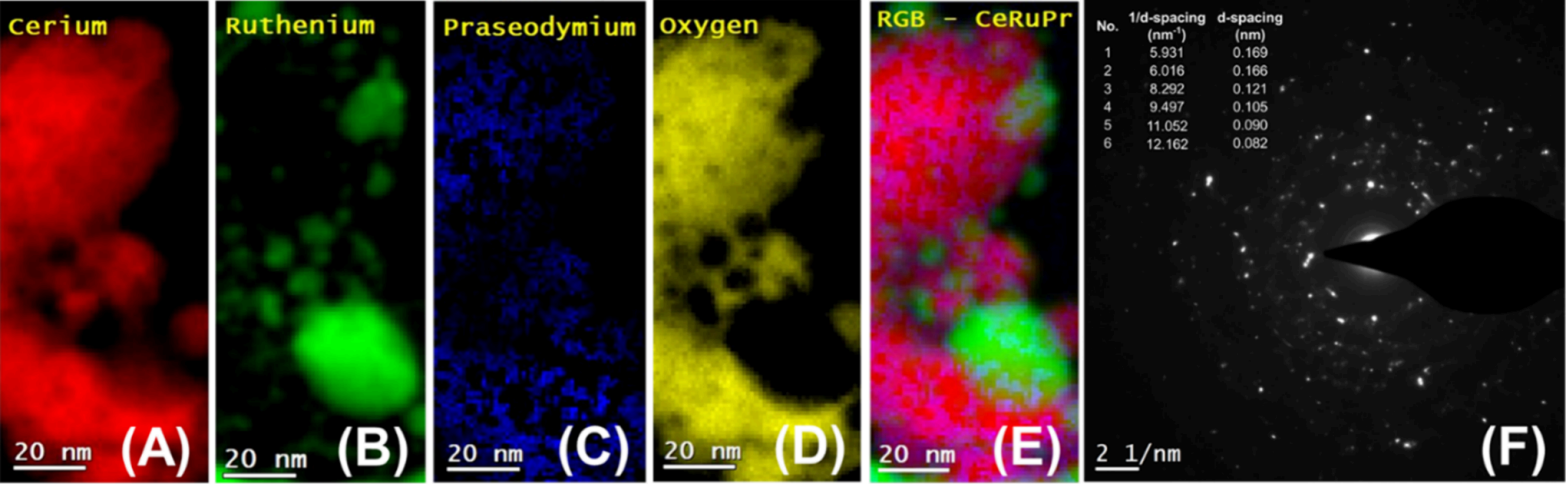
(A–E) STEM-HAADF RGB analysis and (F) selected area electron diffraction (SAED) over the Ru/CePrO catalyst.

**Figure 8:**
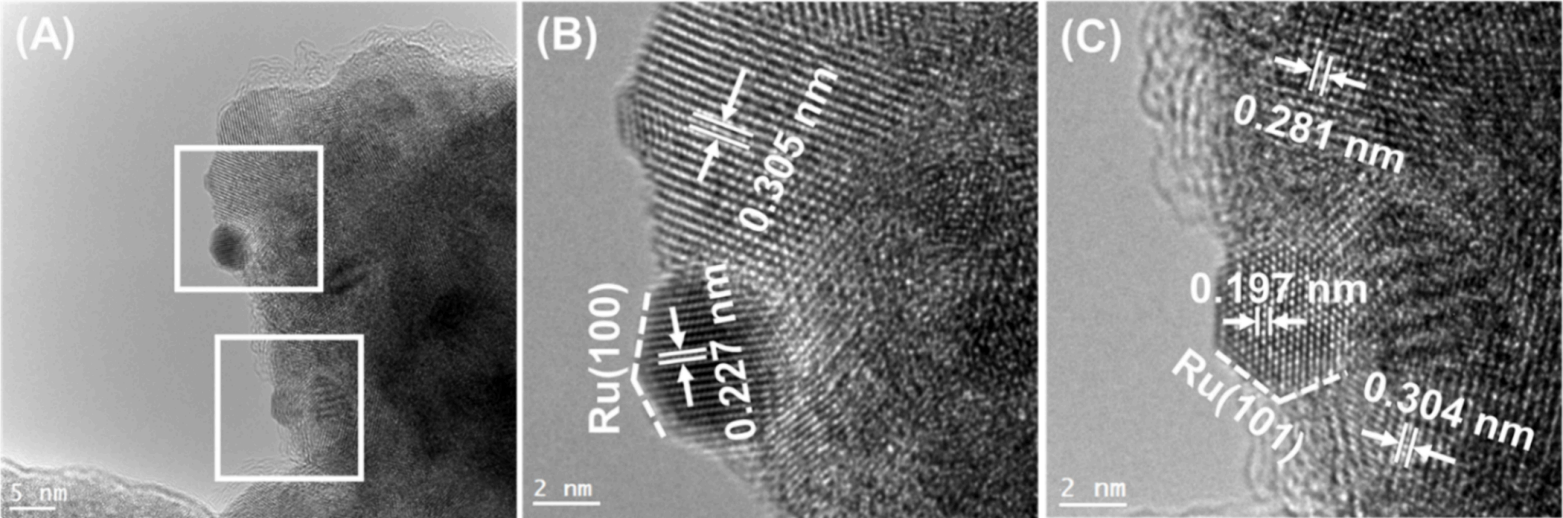
(A–C) HRTEM images of the Ru/HEO catalyst with emphasis on Ru particles at different areas (B) and (C).

**Figure 9:**
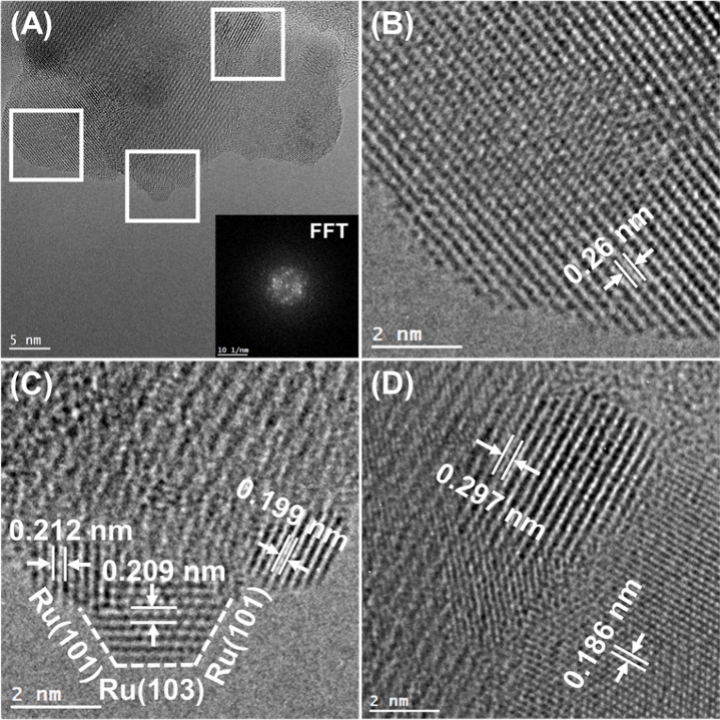
(A) HRTEM images of the Ru/HEO catalyst along with Fast Fourier Transform (FFT) pattern and (B–D) facets analysis.

**Figure 10:**
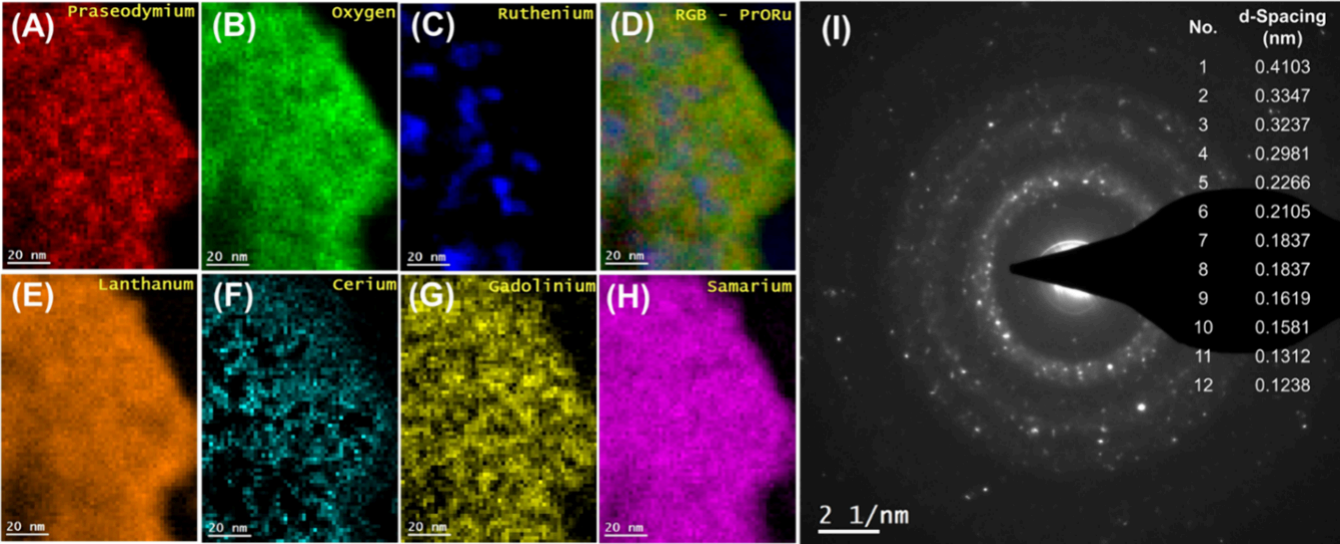
(A–H) STEM-HAADF RGB analysis and (I) selected area electron diffraction (SAED) over the Ru/HEO catalyst.

In a complementary fashion to the H_2_ chemisorption studies, HRTEM studies were performed and the results are presented in Figures 6, 7, 8, 9, and 10 along with their STEM-HAADF Red Green Blue (RGB) and Fast Fourier Transform (FFT) analysis which allows us to comment on the distribution of the elements in the oxide matrix and the exposed facets in the particles, respectively. In particular, in Figures 6 and 7, and Figures 8, 9, and 10 the HRTEM and STEM-HAADF RGB analysis of the Ru/CePrO and Ru/HEO catalysts, respectively, are presented, following their reduction at 650 °C for 2 h. Comparing the HRTEM images in Figure 6 with Figures 8 and 9 it can be concluded that Ru particles grow in a more faceted manner (sharp planes) over the CePrO compared to HEO support. In the Figure 6, lattice fringes with interplanar spacing of 0.196 nm, 0.227 nm can be assigned to the Ru(101) and Ru(100) planes, respectively.

Ammonia synthesis reaction and its rate-determining step, that of N_2_ activation, exhibits high sensitivity in particle shape and faceting.^62^ The facets exposed in the cases of Ru/CePrO and Ru/HEO were confirmed through FFT analysis (Figures 7 and 9 and Tables S5, S6). Regarding the Ru particle size, histogram analysis (Figure S7), based on the HRTEM studies, also showed that Ru particle size is ∼6 and ∼12 nm in the cases of HEO and CePrO-supported catalysts, respectively. These values of particle size are quite in agreement with the H_2_ chemisorption studies above; some overestimation of dispersion can be observed in the chemisorption studies due to the well-known phenomenon of atomic hydrogen spillover in the ceria-based supports. In brief, it is reported by Rarog-Pilecka et al.^63^ that the ammonia synthesis rate increased with the particle size increasing from 0.7 to 4 nm, whereas smaller sizes of Ru crystallites (smaller than 0.7 nm) can be totally inactive; this is linked to the lack of B5 sites. Kim et al. reported that double-stepped Ru(109) sites are more active than the stepped Ru(0001) surface.^64−70^

#### 3.2.6 Catalytic Performance toward NH_3_ Production

**Figure 11:**
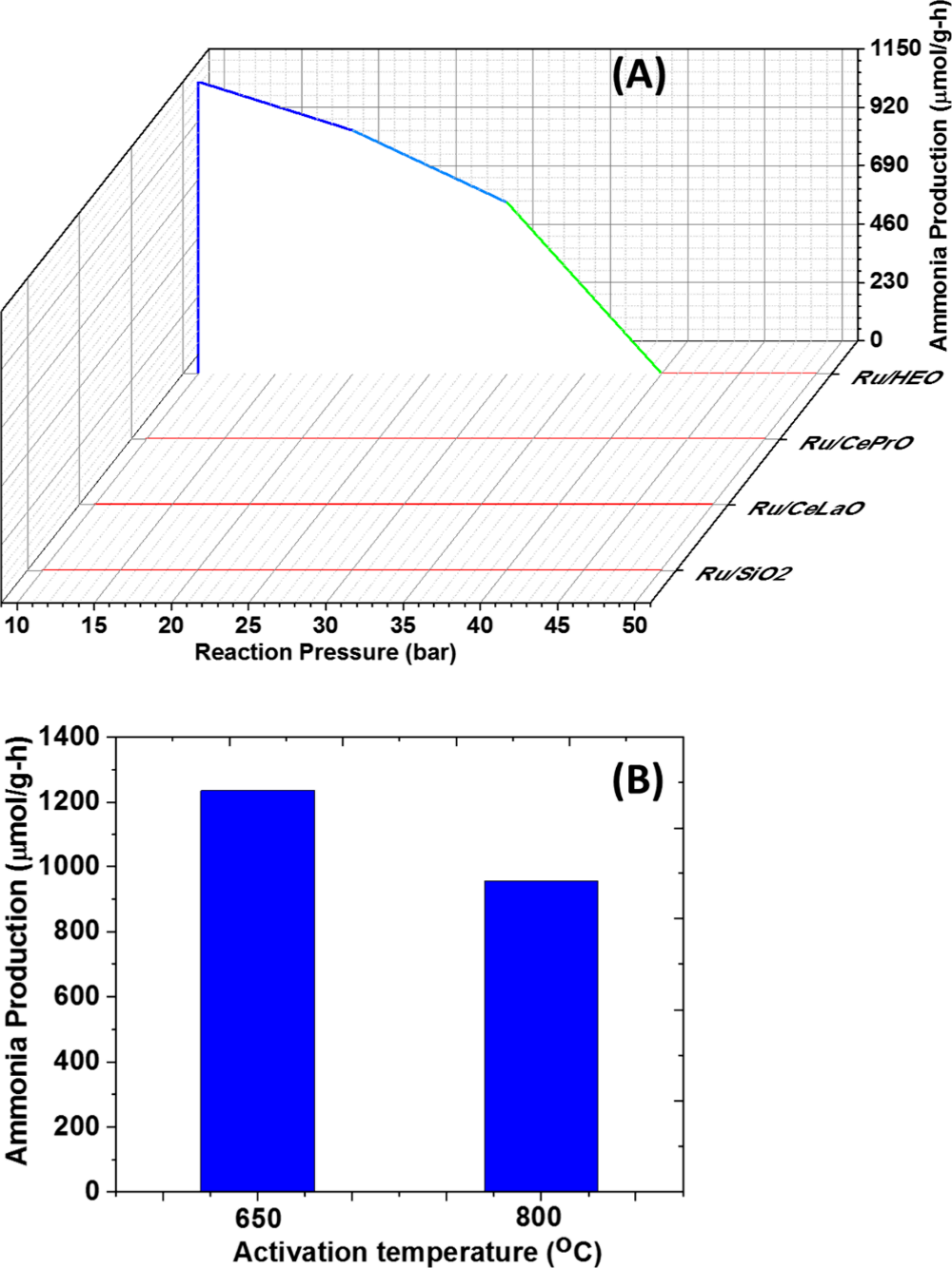
Catalytic production of NH_3_ (A) over Ru-based catalysts of this study in the 10–40 bar pressure range and 400 °C; (B) over the Ru/HEO catalyst at 10 bar, 400 °C following two different activation conditions (650 vs 800 °C).

All the Ru-based catalysts of this study were tested following their reduction at 650 °C for 2h for ammonia production at different reaction pressures (Figure 11A), whereas the best-performing one was evaluated following different activation conditions (Figure 11B). In particular, reaction pressures in the 10–50 bar were used; additionally, the catalysts were subjected to different activation temperatures so to induce different populations of O_v_ sites; namely, reduction at 650 °C (1) and reduction at 800 °C (2). The Ru/HEO catalyst following reduction at 650 °C (catalyst 1, Figure 11A) led to the production of 1236 μmols NH_3_/g-h at a pressure of 10 bar. Increasing the reaction pressure results in a drop in catalytic activity for the Ru/HEO catalyst, whereas the rest of the catalysts presented activity close to zero. Following its activation/reduction to 800 °C for 2 h and keeping the reaction pressure at 10 bar, the NH_3_ production drops to 954 μmols NH_3_/g-h (Figure 11B). It is expected that two major parameters are playing a key role in the measured catalytic activity, as presented in Figure 11 toward ammonia production; Ru particle size as this is controlled by the sintering extent (650 vs 800 °C) and active sites population as this dictated by the Ru particle shape; additionally, it is expected that oxygen vacant sites from the HEO are also a critical activity descriptor. It is well documented that N_2_ dissociation (bond energy 945 kJ/mol) is the rate-limiting step in the NH_3_ production.^68,61^ Under reduction conditions it is expected that electron donation is enhanced from Ru to the π* antibonding orbital of N_2_ facilitating the N_2_ activation.

#### 3.2.7 Intertwined Activity Descriptors

In particular, it has been demonstrated by Jacobsen et al.^61^ that the Ru particle size in the 1.8–2.5 nm range contains the maximum number of B5 sites; the latter are the ones favoring the N_2_ dissociative adsorption. It is believed that the results of Figure 11 are the outcome of two competitive trends; the B5 sites drop in population due to Ru sintering and the O_v_ increase in population due to the reduction conditions (O_v_-650 °C > O_v_-800 °C). The trend observed in NH_3_ production is in agreement with the findings of the ^18^O_2_ transient isotopic isothermal experiments and the population of the surface O_v_ (discussed later). For study completeness, and to ensure that there is no NH_3_/N_2_ impurity that can affect the obtained measurements of our study (overestimate of ammonia production), the above experiments were also run under the same conditions in the absence of N_2_ in the feed; no NH_3_ production was found, thus confirming the lack of ambiguity in our measurements as per the open literature.^69,70^

#### 3.2.8 Ru Metal Particles’ Role

In an effort to untangle the role of Ru particle size on the N_2_ dissociation, it is worth revisiting the recently published report by Yanliang Zhou,^71^ where it is clearly stated that Ru size reduction, in the case of Ru/BaCeO_3_ catalysts, enhances the formation of Ce^3+^ species and oxygen vacancies (O_v_) sites; the latter facilitates the donation of electrons to the Ru sites and thus enhances the N_2_ dissociation step. Additionally, the structure sensitivity of the ammonia synthesis reaction has been highlighted in the study of Peng et al.^72^ at mild conditions; it is manifested by significant changes in catalyst activity at only small structural alterations. In particular, it was reported that the population of corner sites (low coordination sites) increases with dropping Ru particle size with a simultaneous drop of the terrace sites population; additionally, due to the geometric changes (shape and size), the Ru electronic structure is tailored with its size tuning. This causes a reduction to the catalyst work function, ϕ_cat_, which subsequently facilitates the electron donation from the d-orbitals of Ru to the N_2_, enhancing N_2_ activation and N–H bond formation. Isotopic labeling experiments coupled with DRIFTS can be used to trace the size of the pool of the key N-containing intermediates and the mechanism followed (associative vs dissociative); this is important, particularly due to the fact that Ru size can be the crucial factor determining the mechanism too; namely, Ru-based catalysts with particle size >2 nm favor a dissociative pathway,^73^ whereas associative pathway is followed in the case of almost atomically dispersed Ru catalysts.^74,75^ Moreover, smaller Ru size (higher dispersion) can facilitate the H-spillover from Ru to support/interface and induce the H trapping in O_v_-H entities, thus blocking the H-poisoning (parasitic) effect of Ru^76^ which takes place under the reaction conditions. It has to be mentioned that the location (surface/subsurface) and dynamics (clustering) of the O_v_ in trapping H atoms in O_v_-H entities are yet to be determined.

#### 3.2.9 Oxygen Vacancies’ Role

It is noteworthy that O_v_ plays a crucial role and it is almost synonymous with the ceria-based materials’ functionality. However, caution should be exercised when a direct correlation of O_v_ presence and catalytic activity is attempted for reasons commented in what follows and related to O_v_ location and dynamics. Jennifer L. M. Rupp and her collaborators in an enlightening review^77^ discuss the structural arrangements of the O_v_ in the ceria-doped materials (Ce^3+^ = 128 pm, Ce^4+^ = 111 pm). Authors point out that elements/dopants smaller than Gd (Gd^3+^ = 119 pm) favor the placement of the vacancy at the nearest neighbor (NN) site, whereas the elements that are larger than Sm (Sm^3+^ = 96 pm) repel the vacancy from this location. Themselves, Gd and Sm, exhibit neither attractive nor repulsive traits toward vacancies location in the NN site. An opposite trend has been found for the case of the next nearest neighbor (NNN) site, where locking the O_v_ at this site seems never to be preferred. Computational studies show that as the population of NN rare earth ions surrounding the vacancy increases, the defect association energy increases too. Therefore, the local configurations of dopants and oxygen vacancies in doped ceria are of particular importance, yet some details are still not completely understood. Particularly for the N_2_ reduction step, there are many studies trying to unresolve the O_v_ role in the reaction. In the work by Yanliang Zhou^71^ authors investigated the effect of different contents of O_v_ (equivalent to different contents of Ce^3+^ entities) through Bader charge analysis. In the structural models (zero O_v_, 1 O_v_, and 2 O_v_) adopted, the O_v_ content was 0, 3.3, and 6.7%, respectively, giving rise to 2.3–5% O_v_ content in the Ru/BaCeO_2_ catalyst. It was found that the charge donation from Ce^3+^ and O_v_ to the Ru increases as the O_v_ content increases. Oxygen vacancies dictate the charge accumulation to Ru particles, while they can be the drive for mechanism pathway change.^78^ Though, another characteristic of O_v_ that needs to be discussed herein is their clustering at a certain level of doping. Younis et al. report the drop in the catalytic activity of Gd-doped ceria when the dopant level passes 15% where the O_v_ vacancies become immobile due to their clustering, stressing the importance of vacancies dynamics.^58^

### 3.3 Insights into the Oxygen Mobility and Vacancy Formation

**Figure 12:**
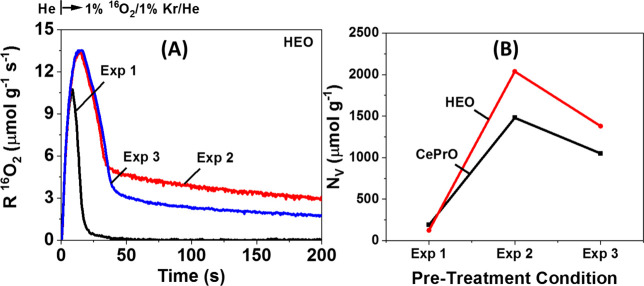
(A) Transient rates of ^16^O_2_ (μmol g^–1^ s^–1^) consumed during transient isothermal oxidation (TIO) at 650 °C, following the different pretreatment conditions, over HEO solid; and (B) amounts of O_2_ consumed (μmol g^–1^) during TIO, over both solids.

To get a thorough understanding of the fine differences in terms of oxygen mobility and vacancy formation of the HEO vs CePrO binary oxide, a ^18^O_2_ transient isotopic isothermal exchange (TIIE) experiment was performed. The transient rates of ^16^O_2_ consumption (μmol g^–1^ s^–1^) obtained during TIO at 650 °C followed by catalyst pretreatment (Exp 1–3) over the HEO solid, are shown in Figure 12A. It can be clearly seen that the rate of oxygen consumed (and its maximum) is lower in the case where no reduction (**Exp 1**) was performed during the pretreatment of the solid, compared to the cases where H_2_ reduction was performed. It is evident that by applying H_2_ reduction after calcination, oxygen vacancies were formed, the amount of which depends on the temperature of reduction. At the initial stage (before maximum), **Exp 2** and **Exp 3** exhibit similar rates, indicating similar amounts of surface oxygen vacancies. Whereas, with time under ^16^O_2_/Kr/Ar/He gas stream, the amount of oxygen vacancies in the bulk is higher in the case where the catalyst reduced at 650 °C, instead of 800 °C. It should be noted that the higher the O_2_ consumption amount, the more oxygen vacancies exist. Figure 12B presents the total amount of O_2_ consumed (N_V_, μmol g^–1^) as a function of pretreatment conditions applied over the two solids. More precisely, independent of the catalyst pretreatment protocol followed, the HEO exhibits more oxygen vacant sites, compared to the CePrO, with an optimum condition being that of catalyst reduction with 5% H_2_/He at 650 °C (Exp 2).

**Figure 13:**
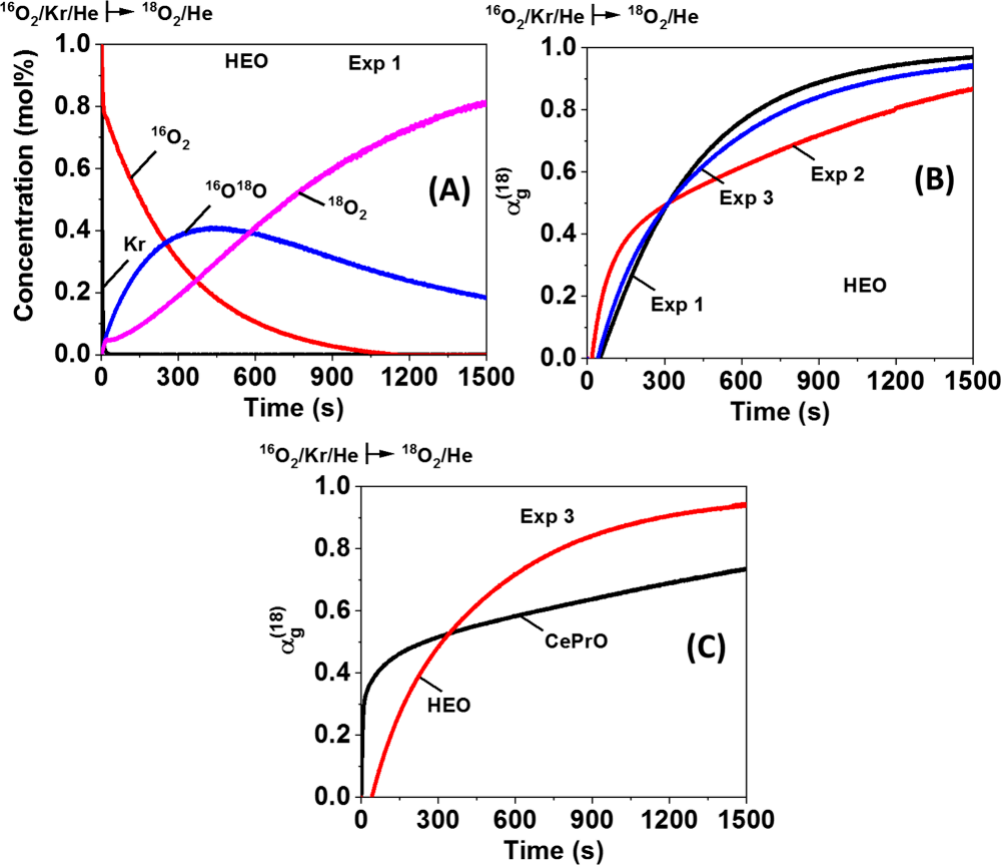
(A) Transient concentration (mol %) response curves of Kr, ^16^O_2_, ^16^O^18^O, and ^18^O_2_ gaseous species recorded during the ^18^O_2_-TIIE experiment at 650 °C, over the calcined HEO; (B)  descriptor as a function of time and pretreatment procedure, estimated during ^18^O_2_-TIIE over HEO; and (C)  descriptor obtained over the both reduced at 650 °C solids.

Figure 13A displays the transient concentration (mol %) response curves of Kr, ^16^O_2_, ^16^O^18^O, and ^18^O_2_ obtained during the ^18^O_2_-TIIE switch from ^16^O_2_/Kr/Ar/He to ^18^O_2_/Ar/He gas mixture at 650 °C, over HEO pretreated via Exp 1. After the switch to the ^18^O_2_/Ar/He, the ^16^O_2_(g) and ^18^O_2_(g) concentrations decrease and increase, respectively, while the formation of ^16^O^18^O(g) starts immediately and passes through a maximum at about 450 s, following by a decreasing tail controlled mostly from the bulk ^16^O diffusion toward the surface. The large difference between ^16^O_2_ decay and that of inert Kr, which lasts for ∼15 s, can be attributed to the surface ^16^O, ^18^O, and ^16^O^18^O exchange on the catalytic surface, considering that the amount of ^16^O exchanged reached the ML = 1 in the first 20 s of the transient. For *t* > 20 s, subsurface and bulk ^16^O diffusion and exchange toward ^16^O^18^O formation is dominant. The total amount of ^16^O^18^O exchanged (N^16^O), estimated via eq 2, was found ∼1.2 times lower in the case of HEO compared to CePrO, while increases by increasing the H_2_ reduction temperature (from 650 to 800 °C) during the pretreatment process (9.75, 10.21, and 11.99 mmol g^–1^ for HEO, respectively, compared to 11.92, 12.02, and 12.48 mmol g^–1^ for CePrO).

The effect of H_2_ reduction temperature on the  descriptor function, over the HEO is illustrated in Figure 13B. It can be seen that different shapes and positions are obtained in the whole transient by varying the catalyst pretreatment (similar results were obtained over CePrO, not shown). Getting more specific, at the initial period after the ^18^O_2_/Ar/He gas switch, where the ^16^O/^18^O exchange on the surface takes place, a steeper response is obvious in the absence of H_2_ pretreatment (Exp 1), indicating a higher *D*_eff_ value,^37^ opposed to the Exp 2 and 3, where H_2_ reduction was applied after catalyst calcination. The latter behavior could be partly attributed to catalyst sintering during reduction, limiting the surface ^16^O/^18^O exchange. However, for prolonged times under the ^18^O_2_/Ar/He gas mixture (*t* > 450 s), it is clear that when the catalyst is reduced at 650 °C, enhances the oxygen diffusion in the bulk, compared to Exp 3 and 1. Based on the above-offered discussion, and comparing the HEO and CePrO pretreated with 5% H_2_ at 800 °C (see Figure 13C), it is rather clear that the former catalyst promotes to a higher extent the surface ^16^O/^18^O exchange (for *t* < 450 s, steeper and lower ), opposed to the bulk oxygen diffusion (*t* > 450 s), which is higher for the latter Ce20Pr catalytic system.

#### 3.3.1 Insights into the Adsorption of N_2_/N Species on Ru/HEO Catalysts

**Figure 14:**
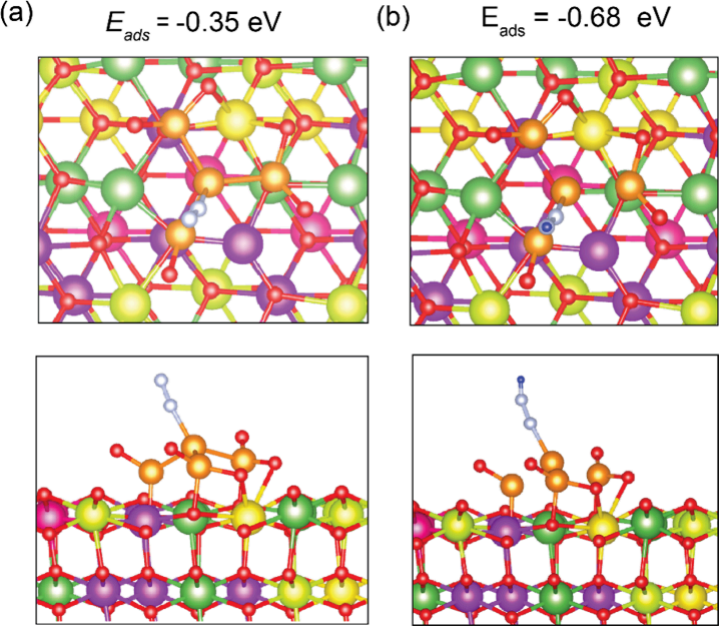
Top and side views of N_2_ Adsorption configurations on Ru4/reduced HEO surface: (a) in absence of hydrogen and (b) in the presence of hydrogen. Gd is shown in purple, La in dark green, Pr in yellow, Sm in pink, Ce in light green, Ru in orange, N in white, H in blue, and O in red.

The understanding of the interaction between the Ru/HEO catalytic surface and N_*x*_ (*x* = 1 or 2) species is expected to avail valuable insights into ammonia synthesis reaction and the experimental results presented above (Figure 13). Therefore, the ab initio studies were performed to examine the catalytic activity of the Ru nanoparticles and HEO support in the adsorption of N_2_ molecules in the presence and absence of hydrogen. Considering the first step for ammonia synthesis is the activation of the N_2_ molecule, the adsorption of the N_2_ molecule on the reduced HEO surface (Ru_4_/HEO) is considered by placing the N_2_ molecule at different sites on the surface consisting of Ru_4_ cluster. The configuration with the minimum energy is considered for further analysis and is shown in Figure 14a. As shown in the figure the N_2_ molecule prefers to bind to the surface over the Ru_4_ cluster. This suggests that the presence of the Ru_4_ cluster facilitates the adsorption of N_2_ molecules on the HEO surface, thereby initiating the first step in ammonia formation. The adsorption energy of the N_2_ molecule is calculated to be −0.35 eV, with a Ru–N bond length of 2.02 Å. The N–N bond length decreased slightly to 1.17 eV compared to the N–N bond length in the gas phase (1.19 Å). For the next step in ammonia synthesis, the adsorption of N_2_ molecules in the presence of H_2_ is analyzed. The adsorption energy of N_2_ further reduces to −0.68 eV in the presence of H_2_ compared to −0.35 eV in the absence of H_2_. The adsorption configuration is shown in Figure 14b. In this step, the N≡N is broken into double bonds, with top N bonded to H with a bond length of 0.89 Å and bottom N bonded to the Ru_4_ cluster with a bond length of 2.02 Å.

**Figure 15:**
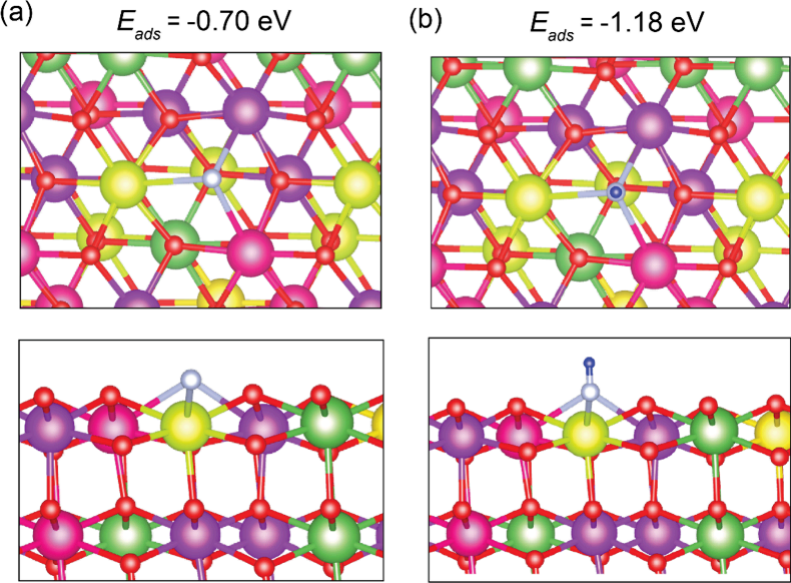
Top and side views of N Adsorption configurations on reduced quinary CeLaPrSmGdO (111) surface: (a) in absence of hydrogen and (b) in the presence of hydrogen. Gd is shown in purple, La in dark green, Pr in yellow, Ce in light green, Sm in pink, H in blue, N in white, and O in red.

It is expected that upon the N_2_ adsorption onto the Ru/HEO catalyst, N_2_ is being dissociated onto Ru sites followed by N≡N bond scission and N atom spillover onto the HEO support. Due to the above findings and including the N spillover effect the binding strength of atomic N on reduced quinary CeLaPrSmGdO (111) surface (i.e., HEO surface) is investigated. The adsorption of atomic N over a reduced HEO surface is studied by placing N over different sites over the reduced HEO surface. Our analysis shows that N over the oxygen-vacant site is most energetically favorable with an adsorption energy of −0.70 eV. The adsorption configuration is shown in Figure 15a. The adsorption of atomic N over the HEO surface is also studied in the presence of H_2_. The adsorption energy of N_2_ over the HEO surface in the presence of H_2_ reduced to −1.18 eV with the H atom bonded to the surface N atom with an N–H bond length of 0.89 Å.

## 4 Conclusions

Briefly, the main conclusions that can be withdrawn from the study are 2-fold. First, in terms of the *understanding of the structure of the HEO* compared to reference binary oxides, CePrO and CeLaO, the following remarks can be pointed out based on the multirange analysis that was performed, XRD (long-range), Raman (medium range) and EXAFS (short range): (1) according to the XRD studies, the HEO reported herein seems to adopt a cubic lattice being isostructural with the CePrO and CeLaO (reference, reducible oxides); (2) EPR and Raman demonstrated that HEO structure bears higher population of oxygen vacancies (O_v_) compared to the binary CePrO under operating conditions (reduction at 650 °C/2 h); though, the abundance of O_v_ varies depending on the length scale probed and the surface/subsurface. In the case of Ru-supported catalysts over the HEO and CePrO supports the quinary one induces strong metal–support interactions (SMSI) and those are manifested through the formation of Ru–O–Ce interfacial species to a greater extent than in the binary oxide (Raman), and the different Ru/Ce and Ru^4+^/Ru^0^ surface intrinsic ratios (XPS); (3) HRTEM studies revealed the effect of support (HEO vs CePrO) on the different mode of growth of Ru particles shape and size (geometrical factor) and demonstrated the role of HEO in getting smaller Ru particle sizes ∼6 nm which are less sharply faceted; (4) In order to induce different degree of O_v_ formation in the oxide lattice, different conditions of H_2_ reduction, after calcination, were applied, driving the formation of O_v_, the amount of which depends on the temperature of reduction (600 vs 800 °C); (5) HEO exhibits more oxygen vacant sites, compared to the reference binary CePrO, yet reducible oxide, with an optimum condition being that of catalyst reduction with 5% H_2_/He at 650 °C due to the extensive sintering happening at 800 °C; (6) The total amount of ^16^O^18^O exchanged (N^16^O), was found ∼1.2 times lower in the case of HEO compared to CePrO, while increases by increasing the H_2_ reduction temperature (650 °C, and 800 °C) during the pretreatment process; (7) HEO promotes in a higher extend the surface ^16^O/^18^O exchange (for *t* < 450 s, steeper and lower ), opposed to the bulk oxygen diffusion (*t* > 450 s), which is higher for the CePrO catalytic system.

Second, these Ru-based catalysts were evaluated toward their activity for ammonia production; the latter being a probe reaction targeting to unveil important features of the above catalysts and guide potential catalysts’ design. In terms of the *role of the HEO in a Ru/HEO catalyst for ammonia production* the following remarks can be made: (1) In the HEO, the higher amount of oxygen vacancies formed at 650 °C, and higher extend of D_eff_, promotes the surface ^16^O/^18^O exchange and leads to surface O_v_ (potential active sites for N_2_ and H_2_ activation); (2) the adsorption of N atom (being spilled over from the Ru) is more favorable at the neighboring site to oxygen vacancy (location of oxygen vacancy) as compared to the one far from the O_v_; (3) The intertwined connection of Ru particle size and population/local environment of surface O_v_ in the HEO facilitates the efficient N_2_ dissociation and thus leads to higher NH_3_ yield (μmol/g-h); (4) The different degree of Ru particles faceting is associated with the variation of B5 sites population and thus the different performance of the catalysts for the reaction at hand.

## Supporting Information Available

Experimental protocol for catalysts characterization that was followed in Raman spectroscopy; X-ray photoelectron spectroscopy; high-resolution transmission electron microscopy (HRTEM), electron paramagnetic resonance (EPR), and H_2_ chemisorption (H_2_-temperature-programmed desorption, TPD); textural analysis results as obtained after N_2_ adsorption at 77 K; SEM microphotographs obtained over the Ru catalysts of this study; EPR spectra obtained at room temperature (298 K) over 6Ru/SiO_2_, 6Ru/CePrO, and 6Ru/HEO catalysts; comparative raman spectra of Ru/CePrO and Ru/HEO catalysts; deconvolution of Ru 3p core-level spectra of Ru/CePrO and Ru/HEO; deconvolution of O 1s core-level spectra of Ru/CePrO, Ru/CeLaO and Ru/HEO catalysts; areas of the O 1s deconvoluted peaks; Ru dispersion and particle size as obtained from the H_2_ chemisorption studies; and FFT analysis of the Ru/HEO catalyst (PDF)
