# Supplementary material for: Evolution of Oxygen Vacancy Sites in Ceria-Based High-Entropy Oxides and Their Role in N2 Activation
Source: ACS Appl Mater Interfaces. 2024 Apr 29;16(18):23038–53. doi: 10.1021/acsami.3c16521 (PMC11082846; doi:10.1021/acsami.3c16521)
Supplement: Supplementary file 1 — am3c16521_si_001.pdf [file am3c16521_si_001.pdf]

SUPPORTING INFORMATION

Evolution of oxygen vacancy sites in ceria-based high-entropy oxides and their role in N_2 activation

Omer Elmutasim[1,2], Aseel G. Hussien[1,2], Abhishek Sharan[2,3], Sara AlKhoori[1,2], Michalis A. Vasiliades[4], Inas Magdy Abdelrahman Taha[3], Seokjin Kim[5], Messaoud Harfouche[6], Abdul-Hamid Emwas[7], Dalaver H. Anjum[2,3], Angelos M. Efstathiou[4], Cafer T. Yavuz[5], Nirpendra Singh[2,3,*], Kyriaki Polychronopoulou[1,2*]

[1]*Mechanical Engineering Department, Khalifa University, P.O. Box 127788, Abu Dhabi, United Arab Emirates*

[2]*Center for Catalysis and Separation (CeCaS), Khalifa University, P.O. Box 127788, Abu Dhabi, United Arab Emirates*

[3] Physics Department, *Khalifa University, P.O. Box 127788, Abu Dhabi, United Arab Emirates*

[4] *Department of Chemistry, Heterogeneous Catalysis Laboratory, University of Cyprus, 1 University Avenue, University Campus, 2109 Nicosia, Cyprus*

[5] *Oxide & Organic Nanomaterials for Energy & Environment (ONE) Laboratory, Advanced Membranes & Porous Materials (AMPM) Center, and KAUST Catalysis Center (KCC), Physical Science & Engineering (PSE), King Abdullah University of Science and Technology (KAUST), Thuwal 23955, Saudi Arabia*

[6]*Synchrotron-light for Experimental Science and Applications in the Middle East (SESAME), Allan, 19252 Jordan*

[7] *Core Labs, King Abdullah University of Science and Technology (KAUST), Thuwal 23955-6900, Saudi Arabia*

*Corresponding authors email:

kyriaki.polychrono@ku.ac.ae,

Nirpendra.singh@ku.ac.ae

Raman. Raman spectroscopy was performed to complement powder XRD and probe the oxygen sublattice and induced structural defects. Raman spectroscopy (Horiba JobinYvon) instrument, green laser (λ = 633 nm) and 50 × objective lens was used. Raman studies were performed over reduced samples (H_2 atmosphere, 650°C, 2h).

XPS. The as-synthesized catalysts were analyzed using XPS in order to assess the effectiveness of the in situ Ru reduction applied during the synthesis. The elemental concentrations and chemical states on the surface were assessed through X-ray photoelectron spectroscopy (**XPS**) using a ThermoScientific™ ESCALAB™ QXi Spectrometer equipped with a monochromated Al Kα X-ray source with a photon energy of 1486.6 eV.

HRTEM. To analyze the samples, Titan 80-300 ST transmission electron microscope (**TEM**) (Thermo Fisher Scientific Inc.) was utilized to carry out TEM analysis. The analysis was performed by operating the microscope at the accelerating voltage of 300 kV. The microscope was set to bright-field (BF) TEM imaging so to acquire images of the samples both at low magnification (LM) and high magnification (HM); this allowed the investigation of the morphology and structure of the nanoparticles (NPs), respectively. Furthermore, the structural analysis of the NPs was conducted using the selected area electron diffraction (SAED) technique. The d-spacings measured from the acquired SAED patterns were compared with the ones obtained through XRD. To determine the distributions of each element in the samples at the nanometer scale, we utilized electron energy loss spectroscopy (EELS) coupled with scanning transmission electron microscopy (STEM). The utilization of EELS generated Ru maps provided an effective means to determine the size of the Ru NPs, compared to using the concentrated Bright-field TEM image, which can be influenced by differences in material thickness.

H_2 Chemisorption (H_2-TPD). The H_2-TPD technique was used to study the dispersion of the supported Ru catalysts. Calcination of the catalyst at 650°C/4 h (static air, furnace) was the first step. Then, 0.1 g of sample was loaded in the U-tube microreactor, and the temperature was increased under He gas flow up to 650°C. At these conditions reduction of the sample took place in hydrogen gas flow (1 bar) at 650°C/2 h, followed by He purge at 650°C until the H_2-TCD signal was stabilized at its background value. The catalyst was

cooled down to 30ºC in He flow; then 30-min of exposure to a 0.5 vol.% H_2/He adsorption gas followed. In order to limit the extent of the H-spillover effect use of lower or higher adsorption times in H_2/He resulted in very similar chemisorption amounts (within experimental error). After H_2 chemisorption, the sample was purged in He flow for 10 min and its temperature was then increased to 700ºC (β = 30ºC min^{-1} , H_2-TPD). The H_2 signal (m/z = 2) was continuously monitored with online thermal conductivity detector (TCD) and converted into concentration (mol%) using a certified gas mixture (0.95 vol% H_2/He).

EPR. The electron paramagnetic resonance (**EPR**) studies were conducted with a Bruker ELEXSYS E500 spectrometer operating at the X band, and using a continuous wave (CW) setup. The spectrometer featured a superhigh Q (ER 4122 SHQ) resonator. In this study the EPR spectra were collected at two temperatures (298 K and 100 K). The microwave frequency of 9.42 GHz with 20 dB microwave attenuation, 5 G modulation amplitude, and 100 kHz modulation frequency were used. The Bruker Xenon software (Bruker BioSpin, Rheinstetten, Germany) was employed for data collection.

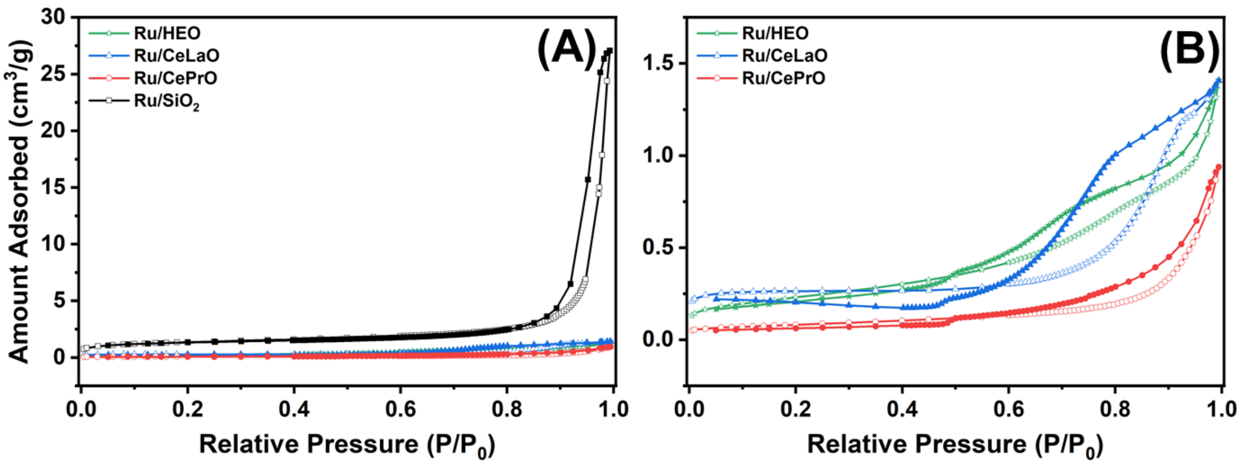

Figure S1: (A-B) N_2 adsorption/desorption isotherms at 77 K over the Ru/HEO, Ru/CeLaO, Ru/CePrO, and Ru/SiO_2.

Table S1: Textural properties of the catalysts in this study.

Catalyst	Surface Area (m^2/g)		Total Pore Volume[b] (cm^3/g)
	Total[a]	External[b]	
Ru/SiO_2	102	80	0.936
Ru/CePrO	6	6	0.032
Ru/CeLaO	18	3	0.048
Ru/HEO	18	17	0.048

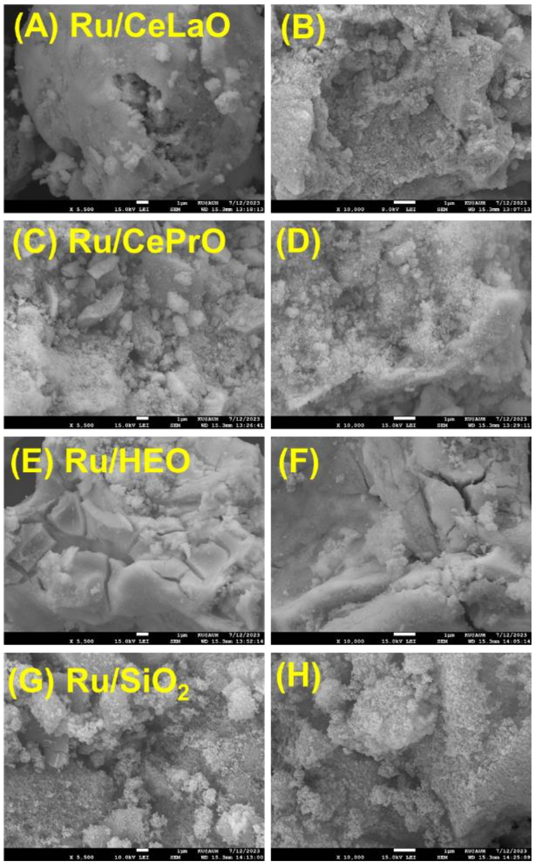

Figure S2: SEM microphotographs obtained over the Ru catalysts of this study.

Table S2: SEM-EDS elemental composition of the catalysts of the present study.

Catalyst	O	Ru	Si	Ce	La	Pr	Sm	Gd
Ru/SiO_2	70.7	0.8	28.5	-	-	-	-	-
Ru/CeLaO	67.2	6.0	-	21.1	5.7	-	-	-
Ru/CePrO	61.6	10.2	-	22.4	-	5.8	-	-
Ru/HEO	66.9	4.3	-	5.3	5.9	6.8	5.5	5.3

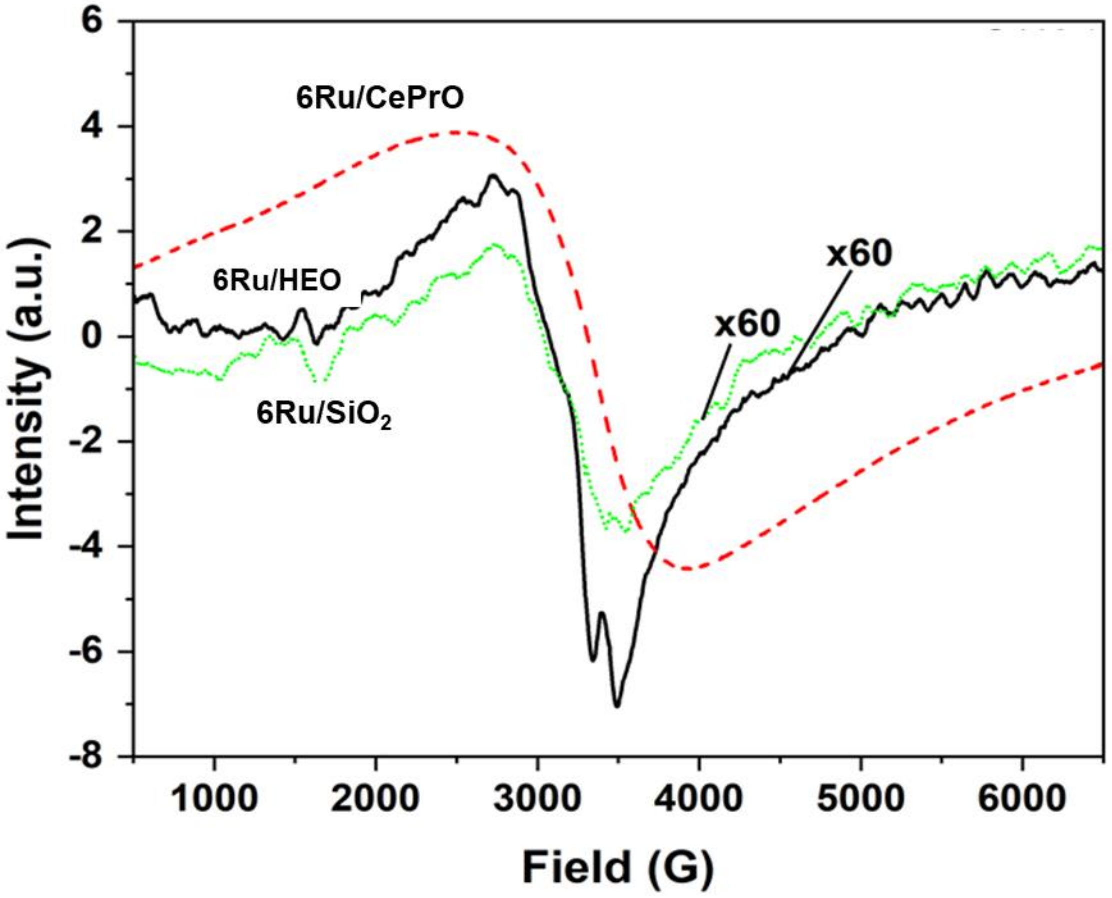

Figure S3: EPR spectra obtained at room temperature (298K) over 6Ru/SiO_2, 6Ru/CePrO, and 6Ru/HEO catalysts.

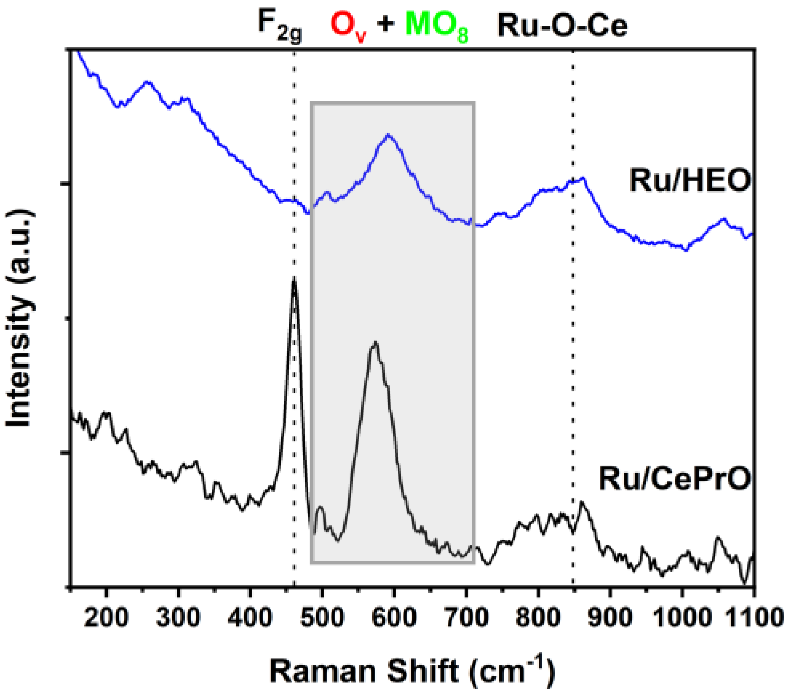

Figure S4: Comparative Raman spectra of Ru/CePrO and Ru/HEO catalysts.

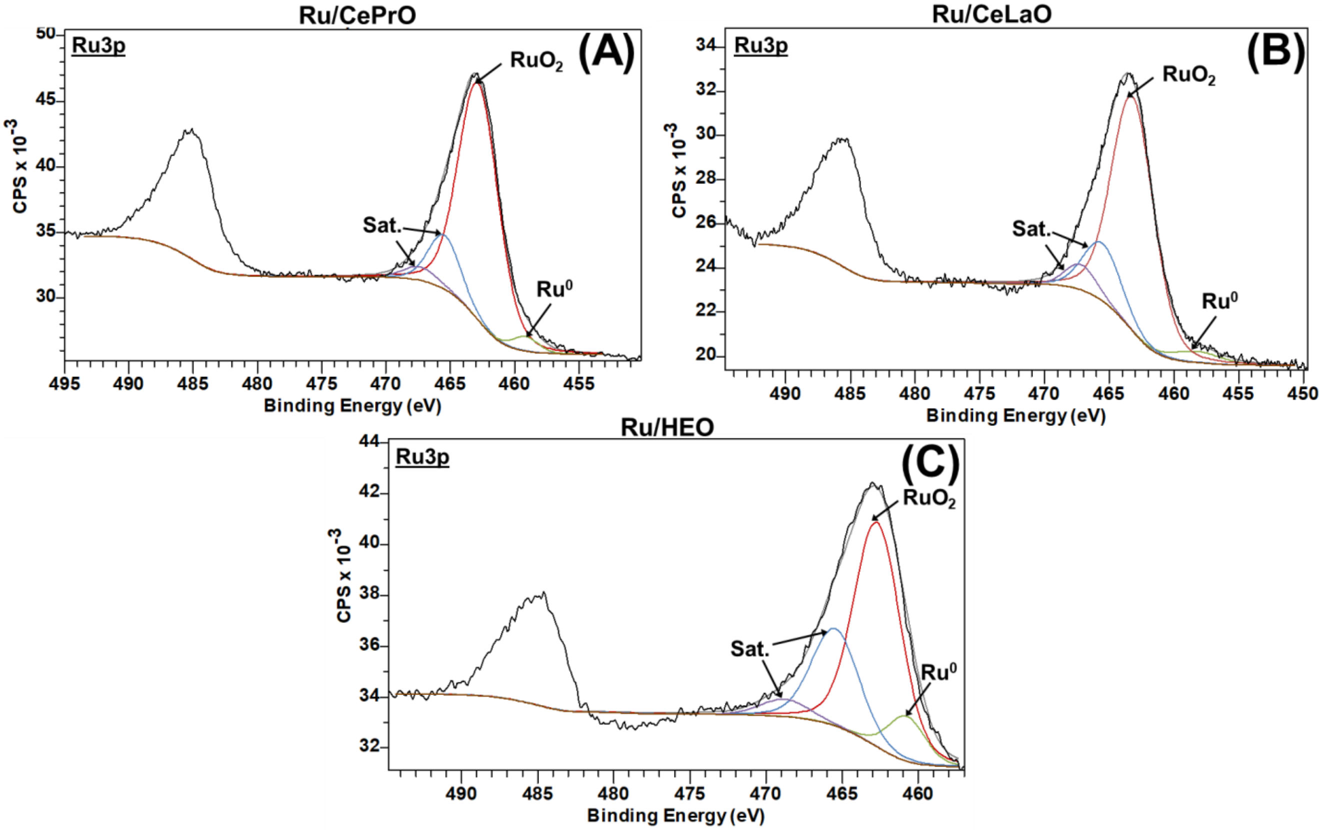

Figure S5: Deconvolution of Ru3p core-level spectra of **(A)** Ru/CePrO and **(B)** Ru/HEO.

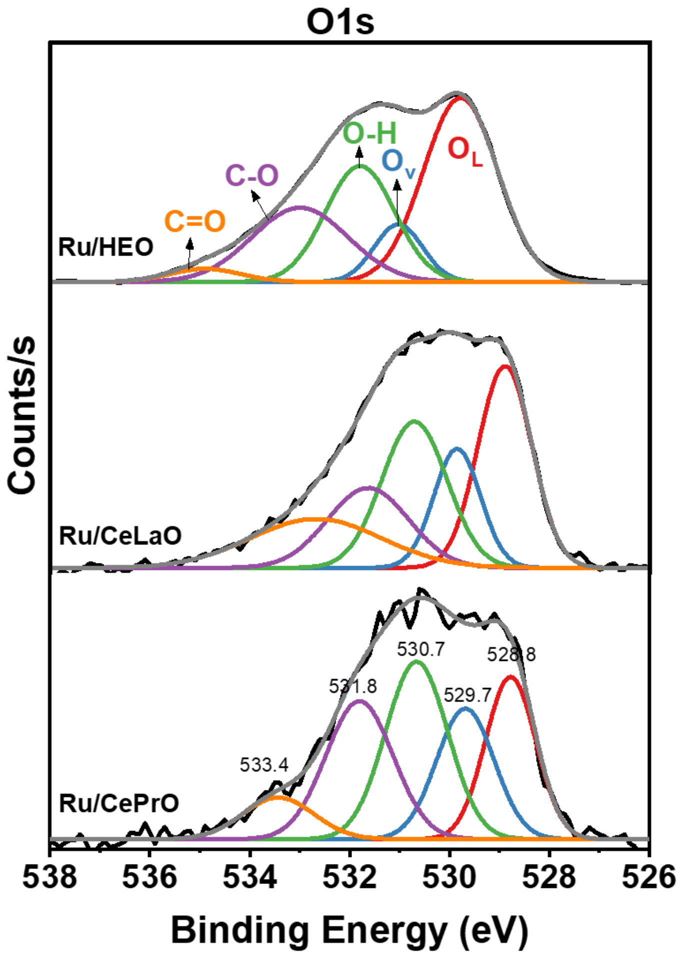

Figure S6: Deconvolution of O1s core-level spectra of Ru/CePrO, Ru/CeLaO and Ru/HEO catalysts.

Table S3. Areas of the O1s deconvoluted peaks.

Catalyst	O_L	O_v	O-H	C-O	C=O
Ru/CePrO	1850	1701	2497	2100	670
Ru/CeLaO	8862	4522	7754	5285	5220
Ru/HEO	79236	16113	46630	41162	5296

Table S4: Ru dispersion and particle size as obtained from the H_2 chemisorption studies.

Catalyst	Dispersion (%)	d_{Ru} (nm)
Ru/SiO_2	17.6	5.7
Ru/CePrO	8.8	11.4
Ru/CeLaO	16.5	6.0
Ru/HEO	23.7	4.2

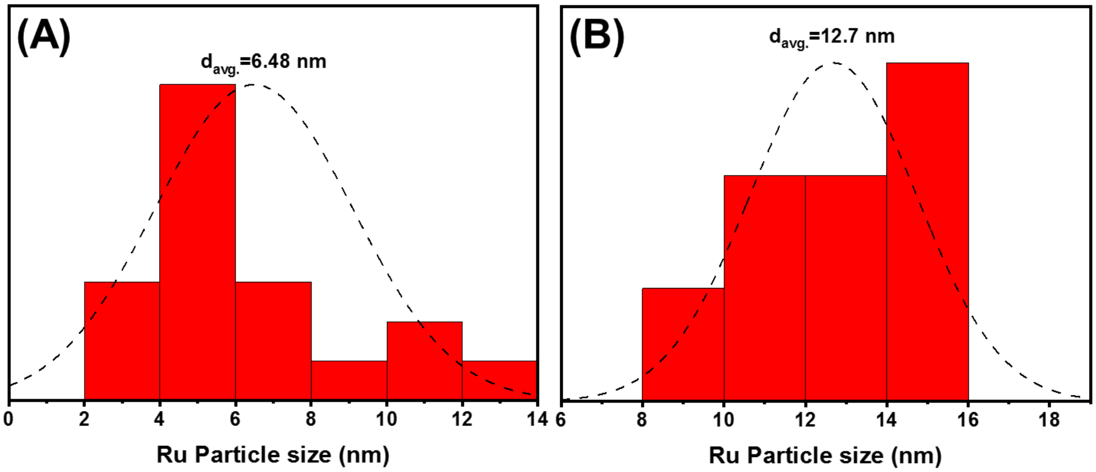

Figure S7: Ru particle distribution analysis based on HRTEM studies **(A)** Ru/HEO; **(B)** Ru/CePrO.

Table S5: FFT analysis of the Ru/HEO catalyst.

Spot#	d-Spacing (nm)	Rec. Pos.(1/nm)	Degrees to Spot 1	Degrees to x-axis	Amplitude
1	0.3238	3.089	0.00	96.76	1024985.63
2	0.2009	4.977	58.86	37.89	1694950.75
3	0.3113	3.212	107.60	-155.64	296588.66
4	0.2013	4.967	121.56	-141.69	1694950.75
5	0.3196	3.129	179.05	-82.29	1024985.63
6	0.2292	4.364	133.15	-36.40	207582.58
7	0.2334	4.285	47.25	144.01	207582.58

Table S6: FFT analysis of the Ru/HEO catalyst.

Spot#	d-Spacing (nm)	Rec. Pos.(1/nm)	Degrees to Spot 1	Degrees to x-axis	Amplitude
1	0.2140	4.672	0.00	155.11	514152.41
2	0.2283	4.379	24.51	179.62	352640.94
3	0.2323	4.305	114.35	-90.54	326704.50
4	0.4533	2.206	172.22	-32.67	947034.13
5	0.4566	2.190	152.63	2.48	331305.91
6	0.2166	4.618	179.96	-24.92	514152.41
7	0.2298	4.351	64.63	90.48	326704.50
8	0.1507	6.636	91.85	63.27	168003.42
9	0.4361	2.293	8.18	146.93	947034.13
